# Natural Selection Affects Multiple Aspects of Genetic Variation at Putatively Neutral Sites across the Human Genome

**DOI:** 10.1371/journal.pgen.1002326

**Published:** 2011-10-13

**Authors:** Kirk E. Lohmueller, Anders Albrechtsen, Yingrui Li, Su Yeon Kim, Thorfinn Korneliussen, Nicolas Vinckenbosch, Geng Tian, Emilia Huerta-Sanchez, Alison F. Feder, Niels Grarup, Torben Jørgensen, Tao Jiang, Daniel R. Witte, Annelli Sandbæk, Ines Hellmann, Torsten Lauritzen, Torben Hansen, Oluf Pedersen, Jun Wang, Rasmus Nielsen

**Affiliations:** 1 Department of Integrative Biology, University of California Berkeley, Berkeley, California, United States of America; 2 Department of Biology, University of Copenhagen, Copenhagen, Denmark; 3 BGI-Shenzhen, Shenzhen, China; 4 Department of Statistics, University of California Berkeley, Berkeley, California, United States of America; 5 Beijing Institute of Genomics, Chinese Academy of Science, Beijing, China; 6 The Graduate University of Chinese Academy of Sciences, Beijing, China; 7 University of Pennsylvania, Philadelphia, Pennsylvania, United States of America; 8 The Novo Nordisk Foundation Center for Basic Metabolic Research, Faculty of Health Sciences, University of Copenhagen, Copenhagen, Denmark; 9 Hagedorn Research Institute, Gentofte, Denmark; 10 Research Centre for Prevention and Health, Glostrup University Hospital, Glostrup, Denmark; 11 Faculty of Health Sciences, University of Copenhagen, Copenhagen, Denmark; 12 Steno Diabetes Center, Gentofte, Denmark; 13 Department of General Practice, University of Aarhus, Aarhus, Denmark; 14 Department of Mathematics, University of Vienna, Vienna, Austria; 15 Faculty of Health Sciences, University of Southern Denmark, Odense, Denmark; 16 Institute of Biomedical Science, Faculty of Health Sciences, University of Copenhagen, Copenhagen, Denmark; 17 Faculty of Health Sciences, University of Aarhus, Aarhus, Denmark; University of Washington, United States of America

## Abstract

A major question in evolutionary biology is how natural selection has shaped patterns of genetic variation across the human genome. Previous work has documented a reduction in genetic diversity in regions of the genome with low recombination rates. However, it is unclear whether other summaries of genetic variation, like allele frequencies, are also correlated with recombination rate and whether these correlations can be explained solely by negative selection against deleterious mutations or whether positive selection acting on favorable alleles is also required. Here we attempt to address these questions by analyzing three different genome-wide resequencing datasets from European individuals. We document several significant correlations between different genomic features. In particular, we find that average minor allele frequency and diversity are reduced in regions of low recombination and that human diversity, human-chimp divergence, and average minor allele frequency are reduced near genes. Population genetic simulations show that either positive natural selection acting on favorable mutations or negative natural selection acting against deleterious mutations can explain these correlations. However, models with strong positive selection on nonsynonymous mutations and little negative selection predict a stronger negative correlation between neutral diversity and nonsynonymous divergence than observed in the actual data, supporting the importance of negative, rather than positive, selection throughout the genome. Further, we show that the widespread presence of weakly deleterious alleles, rather than a small number of strongly positively selected mutations, is responsible for the correlation between neutral genetic diversity and recombination rate. This work suggests that natural selection has affected multiple aspects of linked neutral variation throughout the human genome and that positive selection is not required to explain these observations.

## Author Summary

While researchers have identified candidate genes that have evolved under positive Darwinian natural selection, less is known about how much of the human genome has been affected by natural selection or whether positive selection has had a greater role at shaping patterns of variation across the human genome than negative selection acting against deleterious mutations. To address these questions, we have combined patterns of genetic variation in three genome-wide resequencing datasets with population genetic models of natural selection. We find that genetic diversity and average minor allele frequency are reduced in regions of the genome with low recombination rate. Additionally, genetic diversity, human-chimp divergence, and average minor allele frequency have been reduced near genes. Overall, while we cannot exclude positive selection at a fraction of mutations, models that include many weakly deleterious mutations throughout the human genome better explain multiple aspects of the genome-wide resequencing data. This work points to negative selection as an important force for shaping patterns of variation and suggests that there are many weakly deleterious mutations at both coding and noncoding sites throughout the human genome. Understanding such mutations will be important for learning about human evolution and the genetic basis of common disease.

## Introduction

A substantial amount of effort in human population genetics has been aimed at understanding how natural selection operates in the human genome. However, we lack a basic understanding of the importance of positive natural selection versus negative selection at shaping overall patterns of genome variation. Thus far, most of the attention has been aimed at locating genes that have been under positive selection [1]–[19]. These studies have identified several hundred candidates throughout the genome that may have been affected by positive natural selection. However, fewer studies have attempted to gauge the prevalence of positive natural selection in the human genome. Those that have attempted have come to very different conclusions. Several studies suggested that positive selection may be common, with around 10% of the genome having been affected by a recent selective sweep [9], [10], [14], [16]. Other studies argued that selective sweeps were less common [20], [21]. Finally, some have estimated that approximately 10%, but perhaps up to 40%, of nonsynonymous human-chimp differences have been fixed by positive natural selection [22], [23]. Thus, there is little consensus regarding the importance of positive natural selection at shaping patterns of variability.

Additionally, the role of negative selection at shaping broad patterns of genetic variation across the genome needs to be clarified. Many studies have suggested that nonsynonymous mutations and mutations in conserved noncoding sequences are weakly deleterious but may persist in the population due to genetic drift and other demographic phenomena [22], [24]–[32]. The effect that these weakly deleterious mutations have on nearby patterns of genetic variation remains unclear. Furthermore, the importance of negative versus positive selection at shaping overall patterns of variation also remains ambiguous.

If natural selection (either positive or negative) is common in the genome, it should affect patterns of genetic variation at linked neutral sites across the genome [33], [34]. Selection may alter genetic variation in different ways. We review these ways, discuss the empirical evidence for these effects, and highlight open questions that our study seeks to address.

First, selection may generate a correlation between levels of neutral diversity and recombination rate [35], [36]. This can occur under models with strong positive selection (selective sweeps) or negative selection acting on many deleterious mutations (background selection). Selective sweeps remove genetic diversity at linked neutral sites [33], [37]. In a region of the genome with a low recombination rate, a large length of sequence will have the same genealogy as the selected site. As such, the selective sweep will remove neutral variation over a larger portion of the sequence in low recombination rate regions than in regions with higher recombination rates. Background selection against deleterious mutations can also generate this correlation [34], [38]–[41]. Chromosomes carrying many deleterious mutations will be rapidly eliminated from the population. Any neutral variation linked to the deleterious mutations will also be eliminated from the population. This model predicts reduced variability in regions of the genome with low recombination rate because, as with the case of a selective sweep, a larger portion of the chromosome will share the same genealogy as the selected site(s) in regions of low recombination rather than in high recombination. Several studies have searched for a correlation between diversity and recombination rate in humans. Early studies based on a small number of genes came to conflicting conclusions. Nachman et al. [42], [43] found a significant correlation between diversity and recombination rate, but found no correlation between divergence and recombination rate, suggesting the effects of natural selection. Hellmann et al. [44], examining a different dataset, found that the correlation between diversity and recombination rate disappeared after correcting for human-chimp divergence. They suggested that recombination may be mutagenic and that the original correlation was driven by co-variation of mutation and recombination rates. Another study found that microsatellite diversity was not correlated with recombination rate [45]. More recent studies on larger datasets have found significant correlations between diversity and recombination rate [46]–[48]. These studies have found that the correlation between human diversity and recombination rate persists after controlling for human-chimp divergence. While this is suggestive of the effects of natural selection, important features of this correlation have yet to be characterized. For example, if natural selection is primarily driving the correlation, the correlation ought to be stronger in genic regions of the genome than in non-genic regions, because functional sites near genes are the most likely targets of selection. This feature has yet to be explored.

Second, natural selection may generate a correlation between the allele frequency distribution and recombination rate. Specifically, models of selective sweeps predict a skew toward an excess of low-frequency single nucleotide polymorphisms (SNPs) near the target of selection [49]–[51]. Following the same logic as above, a larger region of the genome will be affected in areas with lower recombination rates, thus generating a correlation between allele frequency and recombination rate. The effect of background selection on allele frequencies is less clear. Simulation studies have suggested that intermediate strengths of background selection, especially in regions of low recombination, can generate a skew toward an excess of low-frequency SNPs [34], [38], [52]–[58]. Most of the analytical formulae that describe background selection model the process as a reduction in effective population size, which does not predict a skew of the frequency spectrum ([34], [38]–[41], but see Santiago and Caballero [59]). Consequently, it has been argued that the effect of background selection on the frequency spectrum is rather weak, and as such, a skew toward low-frequency SNPs is more indicative of positive, rather than background selection [60]–[66]. It is unclear whether there is a correlation between allele frequency and recombination rate in the human genome, though several small studies have found suggestive evidence [6], [67]. Furthermore, it is unclear which models of selection may be compatible with such a correlation.

Third, if selection is common, it ought to primarily affect patterns of genetic variation near genes because genes are the likely targets of selection. Several studies have found that human-chimp divergence and human diversity were reduced near genes, suggesting the importance of selection at shaping overall patterns of variability throughout the genome [67]–[69]. It is less clear whether there is a skew toward low-frequency alleles near genes.

Fourth, pervasive positive natural selection may generate a negative correlation between nonsynonymous divergence and levels of neutral genetic diversity ([70]–[77] and reviewed in [78]). The reason for this is that selective sweeps acting on amino acid changing mutations generate nonsynonymous fixed differences between species. Regions of the genome that have been affected by these sweeps will likely also have reduced neutral polymorphism, thus generating the negative correlation between these two quantities. It is unclear whether such a correlation can be generated in the absence of positive selection and how strong the correlation might be under various models of positive selection.

Here we further investigate these issues by studying patterns of genetic variation in three different genome-wide genetic variation datasets obtained from resequencing European individuals. We find that levels of diversity are positively correlated with recombination rate and negatively correlated with genic content. Minor allele frequency is also positively correlated with recombination rate and negatively correlated with genic content. Using simulations, we show that these correlations are best explained by a model where many sites are under weak negative selection. Models with numerous selective sweeps on nonsynonymous mutations predict too strong a negative correlation between neutral polymorphism and nonsynonymous divergence. Though not required to explain the data, some smaller fraction of sites may be under positive selection. Overall, this work points to the importance of weak negative selection at shaping patterns of variation throughout the human genome.

## Results

### Summarizing genomic patterns of variation

We analyzed genomic patterns of polymorphism from three genome resequencing datasets. First, we analyzed low-coverage next-generation sequence data obtained from an exome-capture study of 2,000 Danish individuals. Due to the non-specificity of the exome-capture arrays, portions of the genome outside of the targeted regions were sequenced, but at lower coverage. Given the shallow sequencing depth across most of the genome (roughly 0.1× per individual), it would be impossible to infer genotypes for each individual with any appreciable accuracy. Instead, we implemented a statistical approach to estimate the population allele frequency of a SNP using the counts of different nucleotides at a particular site in the genome (see Materials and Methods for a detailed description). When combining reads across all individuals, approximately 30–40% of the genome had a sequencing depth of at least 100 reads. We estimated the minor allele frequency (MAF) for all of these sites with a depth of at least 100 reads. Those sites with an estimated MAF>5% were considered to be SNPs in this dataset and were used for subsequent analyses. We used this conservative cut-off because of the difficulties in reliably estimating allele frequencies of rare alleles in low-coverage data [79].

In order to verify patterns found in our low-coverage resequencing dataset, we also analyzed two other complementary datasets. One dataset consisted of six European genomes that were sequenced to higher coverage (denoted “higher coverage,” see Materials and Methods for details). The other dataset consisted of five genomes from Utah residents with ancestry from northern and western Europe (abbreviated CEU) and one genome from a Toscan individual sampled from Italy (abbreviated TSI) sequenced to high coverage by Complete Genomics (denoted “CGS,” see Materials and Methods). Summaries of genetic variation were positively correlated across the three datasets (Figure S1 and Figure S2). Due to the stochasticity of the evolutionary process, even with perfect data, patterns of polymorphism will not be perfectly correlated across different datasets.

To analyze correlations between different summaries of polymorphism and other genomic features, we divided the genome into non-overlapping 100 kb windows (see Materials and Methods for further details). Within each window, we tabulated the number of SNPs, average MAF, number of human-chimp differences, GC content, recombination rate (as estimated from the high-resolution deCODE map [80]), fraction of each window where sequencing data was available, and the fraction of the window that overlaps with a RefSeq gene. Since we wanted to examine the indirect effects of natural selection due to linkage, rather than assess the effects of natural selection on the selected sites themselves, all of our analyses removed the roughly 5% of the genome that was most conserved across species (i.e. the phastCons regions [81], see Materials and Methods). These were the regions most likely to be directly under negative selection in the human genome [81]. We then assumed that the remaining sequence that we analyzed was selectively neutral. Because many of the genomic features were correlated with each other (Table S1, Table S2, Table S3), we performed partial correlation analyses to remove the effects of possible confounding variables. The partial correlation can be thought of as the correlation between two variables when one or more other confounding variables are held constant. We used partial correlations, rather than a full multivariate analysis, because the partial correlations have a simpler biological interpretation and have been used in other recent evolutionary studies [82].

### Correlation between neutral polymorphism and recombination rate

We found a strong positive correlation between the number of SNPs in a window and the recombination rate of the window (Spearman's 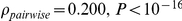, Table S1) when looking at the low-coverage data. We also observed a strong correlation between the number of human-chimp differences within a window (*d*) and recombination rate (Spearman's 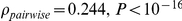, Table S1). When scaling diversity by divergence (i.e. dividing the number of SNPs per covered base within a window by the number of human-chimp differences) to potentially account for differences in mutation rate across the genome, we still found a strong correlation between scaled SNP diversity (defined here as *S_norm_*) and recombination rate (Spearman's 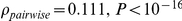, Table 1, Table S1). In particular, regions of the genome with low rates of recombination (i.e. <0.5 cM/Mb) had especially low levels of polymorphism. The rate of change of *S_norm_* was less dramatic over the rest of the range of recombination rates.

**Table 1:** Summary of the correlation coefficients (Spearman's 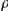) for the three datasets.

Dataset	Correlation type	*S_norm_* vs. recombination rate^a^	MAF vs. recombination rate^a^	*S_norm_* vs. genic content^b^	MAF vs. genic content^b^
Low-coverage	Pairwise correlation	0.111****	0.062****	−0.039***	−0.012
	Partial correlation	0.117****	0.042***	−0.056***	−0.018*
Higher− coverage	Pairwise correlation	0.209****	0.086****	−0.033***	−0.043***
	Partial correlation	0.173****	0.046***	−0.076****	−0.035***
CGS	Pairwise correlation	0.200****	0.101****	−0.040***	−0.040***
	Partial Correlation	0.188****	0.066****	−0.077****	−0.020*

a Partial correlation controls for human-chimp divergence, GC content, genic content, and coverage (the number of neutral bases covered by sequencing data).

b Partial correlation controls for human-chimp divergence, GC content, recombination rate, and coverage (the number of neutral bases covered by sequencing data).

*P<0.05.

**P<0.001.

***P<10^−5^.

****P<10^−16^.

We also found a positive correlation between *S_norm_* and recombination rate when analyzing the higher-coverage and CGS datasets (Spearman's 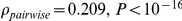, Table 1, and Table S2 for the higher-coverage data; Spearman's 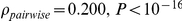, Table 1, and Table S3 for the CGS data). The correlation was even stronger than that observed in the low-coverage data. We discuss several possible reasons for this difference in the Discussion section. Nevertheless, the fact that we found the correlation in all three datasets strongly argues that it is a true biological correlation and not an artifact due to biases in the low-coverage Danish data. The correlation between *S_norm_* and recombination rate remained significant even after controlling for GC content, *d*, the number of neutral bases covered by sequencing data, and the fraction of genic bases within a window (Table 1), suggesting that these factors cannot completely explain this correlation. Further, the average number of pairwise differences per window normalized by *d* was also positively correlated with recombination rate in both datasets (Table S2 and Table S3).

If natural selection is responsible for this correlation between *S_norm_* and recombination rate, it may be stronger in genic regions of the genome than in non-genic regions. The reason for this is that, all else being equal, genic regions will likely experience more natural selection than non-genic regions. Non-genic windows were defined to be those that did not overlap with a RefSeq transcript. Genic windows were those where at least half the window overlapped with a RefSeq transcript.

**Figure 1:**
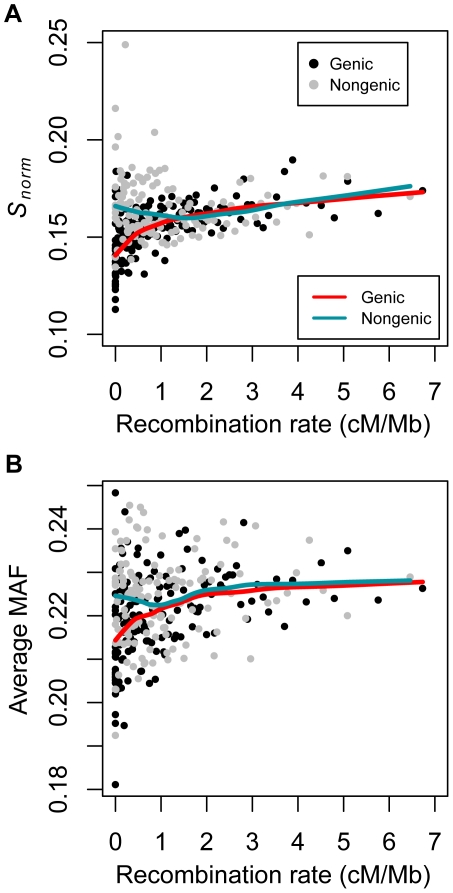
Correlations between summaries of genetic variation and recombination rate in the low-coverage dataset dividing the data into genic and non-genic windows (see text). (A) Number of SNPs per covered base divided by human-chimp divergence (*S_norm_*) versus recombination rate. (B) Average minor allele frequency versus recombination rate. Red and green lines denote the lowess curves fit to the two variables for genic and non-genic windows, respectively. Black points denote genic windows while gray points denote non-genic windows. Each point represents the average statistics computed over 50 100 kb windows. The windows were sorted by recombination rate prior to binning. Note that several outlier data points fell outside the plotting area.

Indeed, the correlation was significantly stronger in genic windows than in non-genic windows in all three datasets (*P*<0.0001 by permutation test, Figure 1, Table 2, Figure S3, and Figure S4). This pattern holds even after controlling for confounding variables using a partial correlation analysis. Inspection of the lowess lines in Figure 1A illustrates the differences between the correlation in genic and non-genic regions. In genic regions with low recombination rates (<0.5 cM/Mb), there is a sharp decrease in *S_norm_*. However, non-genic regions with low recombination rates did not show such a pronounced decrease in *S_norm_* (Figure 1A). One concern with these analyses is that the low-coverage dataset was an exome resequencing dataset and the exome-capture process may have resulted in systematic differences between genic and nongenic regions. However, we found the same pattern in the higher-coverage dataset and the CGS dataset, which were not targeted toward genes or exons (Figure S3 and Figure S4). This argues that the differences between genic and non-genic regions were not due to systematic biases in the data, but rather to inherent differences between genic and non-genic regions of the genome.

**Table 2:** Summary of correlation coefficients (Spearman's 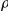) for the three datasets divided into genic and non-genic windows.

Dataset	Correlation type	Window type	*S_norm_* vs. recombination rate^a^	MAF vs. recombination rate^a^
Low coverage	Pairwise correlation	Genic	0.175****	0.073***
		Nongenic	0.028*	0.042**
	Partial correlation	Genic	0.185****	0.029*
		Nongenic	0.050**	0.033*
Higher coverage	Pairwise correlation	Genic	0.250****	0.123****
		Nongenic	0.168****	0.043**
	Partial correlation	Genic	0.209****	0.063***
		Nongenic	0.132****	0.031*
CGS	Pairwise correlation	Genic	0.241****	0.123****
		Nongenic	0.154****	0.074***
	Partial correlation	Genic	0.227****	0.065***
		Nongenic	0.138****	0.055***

a Partial correlation controls for human-chimp divergence, GC content, and coverage (the number of neutral bases covered by sequencing data).

*P<0.05.

**P<0.001.

***P<10^−5^.

****P<10^−16^.

### Correlation between average MAF and recombination rate

We then examined the correlation between average MAF within a window and recombination rate (Table 1 and Table S1) in the low-coverage data. We found a weak, but statistically significant, positive correlation between these two variables (Spearman's 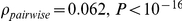). In regions of low recombination, there was a skew toward lower average MAF. The correlation remained significant even after controlling for GC content, *d*, the number of neutral bases covered by sequencing data, and genic content, suggesting that it cannot be completely explained by these other factors (Spearman's 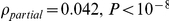, Table 1). Finally, we also found a positive correlation between average MAF and recombination rate in the higher-coverage and the CGS data (Table 1, Table S2, Table S3), again suggesting that it was not due to biases in estimating SNP frequencies from low-coverage data. A different summary of the frequency spectrum, Tajima's *D* [49], also showed a correlation with recombination rate (Table S2 and Table S3), indicating that this correlation was not sensitive to the summary of the frequency spectrum employed.

However, no clear pattern emerged when testing whether the correlation between average MAF and recombination rate was stronger in genic versus non-genic regions. For all three datasets, the pairwise correlation between average MAF and recombination rate was higher in genic regions than non-genic regions (*P*<0.05, by permutation test, Figure 1B, Figure S3B, Figure S4B, Table 2). In the higher-coverage dataset, genic regions showed a stronger correlation between MAF and recombination rate than non-genic regions even after controlling for GC content, *d*, and the number of bases covered by sequencing data using a partial correlation analysis (*P*<0.02 by permutation test, Table 2). However, after controlling for the confounding variables, there was little difference in the partial correlation coefficients between genic and non-genic regions in the low-coverage and the CGS datasets (Table 2). Thus, there was no clear evidence suggesting that the correlation between MAF and recombination rate was stronger in genic than non-genic regions of the genome. This may not be surprising because this correlation was quite weak, making it difficult to detect subtle changes in its strength across the genome.

### Diversity, MAF, and divergence in relation to genes

If natural selection affects patterns of genetic variation across the genome, *S_norm_*, average MAF, and *d* may be reduced in windows of the genome that contain more genic bases. These patterns would be expected if most of the selection in the genome occurs near genes, rather than in intergenic regions.

Indeed, in all three datasets, we found a negative correlation between *S_norm_* and the fraction of bases within a window that overlapped with a RefSeq transcript (Table 1). In other words, windows with a higher genic content tended to have fewer SNPs. These correlations became stronger when controlling for *d*, recombination rate, the fraction of the window with sequencing coverage, and GC content (Table 1).

There was a weak, but significant, negative correlation between MAF and fraction of bases that overlapped with a RefSeq transcript in all three datasets examined (Table 1). Windows with a higher genic content tended to have lower average MAF than windows with lower genic content. In the low-coverage and higher-coverage datasets, the correlation became stronger when controlling for *d*, recombination rate, the fraction of the window with sequencing coverage, and GC content (Table 1).

Finally, we found a very strong negative correlation between *d* and the fraction of genic bases within a window (Spearman's 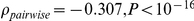, Table S1, Table S2, Table S3). These results were in agreement with those from a study [67] which found reduced diversity and divergence near genes even after removing the regions of the genome most conserved across species (i.e. the phastCons elements).

### Neutral diversity and nonsynonymous divergence

We next tested whether there was a correlation between *S_norm_* and the number of nonsynonymous human-chimp differences within a window (*D_N_*). A negative correlation between these two variables has been interpreted as evidence of selective sweeps across the genome ([70]–[77] and reviewed in [78]). When tabulating *D_N_*, we did not remove sites which were conserved across species. We observed weak negative correlations between *S_norm_* and *D_N_* as well as between *S_norm_* and the number of synonymous human-chimp differences (*D_S_*) for several of the datasets (Table S4). However, when we normalized *D_N_* by the number of nonsynonymous sites per window (the normalized value is called *d_N_*) or used a partial correlation analysis to control for the number of nonsynonymous sites per window, none of the datasets showed a significant negative correlation (Table S4). The same was true for synonymous human-chimp differences.

Haddrill et al. [77] suggested that a negative correlation between *S_norm_* and *d_N_* may be more apparent in genes with elevated *d_N_*. Thus, we also tested for a correlation between *S_norm_* and *d_N_* using only the windows in the 90^th^ percentile of *d_N_*. In general, the values of Spearman's 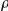 were more negative in this subset of the data than when analyzing the entire dataset (Table S5). For example, in the CGS data, 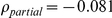 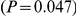 when controlling for *d*, GC content, recombination rate, the number of nonsynonymous sites, and the fraction of the window with sequencing coverage. However, *S_norm_* was also negatively correlated with *d_S_* in the windows in the 90^th^ percentile of *d_S_* (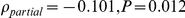, controlling for *d*, GC content, recombination rate, the number of synonymous sites, and the fraction of the window with sequencing coverage). The fact *d_S_* showed a similar negative correlation with *S_norm_* as *d_N_* did, combined with the fact that synonymous sites are usually assumed to be neutrally evolving in humans, suggested that these correlations may have been driven by a neutral process, rather than positive selection. One possibility was that the recent fixations of neutral synonymous or nonsynonymous mutations led to a decrease in neutral diversity, as suggested by earlier theoretical work [83]. As such, regions with high *d_N_* (or high *d_S_*) would have lower *S_norm_*, generating the negative correlation. Overall, these results suggest that regions of the genome that have more nonsynonymous human-chimp differences do not have lower levels of neutral polymorphism, beyond the reduction in diversity already expected in genic regions of the genome or surrounding neutral fixations.

### Correlations predicted by various population genetic models

**Figure 2:**
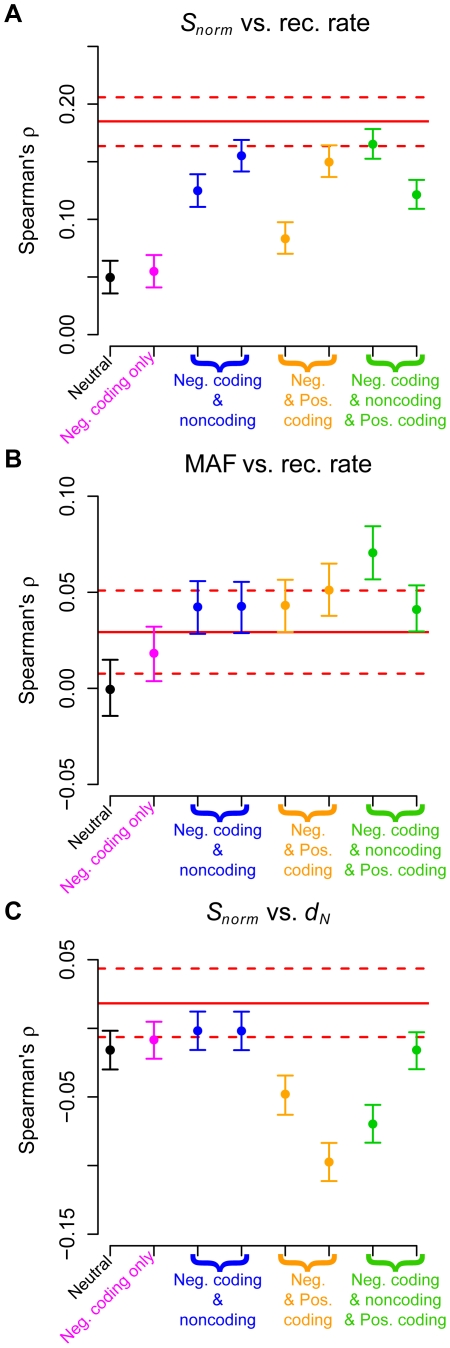
Comparison of Spearman's 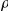 for genic regions with the expected values based on forward simulations for the low-coverage dataset. (A) Number of SNPs per covered base divided by human-chimp divergence (*S_norm_*) versus recombination rate. (B) Average minor allele frequency versus recombination rate. (C) Number of SNPs per covered base divided by human-chimp divergence (*S_norm_*) versus human-chimp nonsynonymous divergence (*d_N_*). The red solid lines denote the point estimates from the genic regions in the low-coverage data. The dotted lines represent 95% confidence intervals obtained by bootstrapping. Black points denote a model with no selection and pink points a model where negative selection acted only on nonsynonymous mutations. Blue points denote models where both nonsynonymous and some intronic sites were subjected to negative selection. Orange points denote models where most nonsynonymous mutations were negatively selected, but some were positively selected. Green points denote models where nonsynonymous and some intronic mutations were subjected to negative selection, but a fraction of nonsynonymous mutations were positively selected. See Table S6 for a more detailed description of the different models of selection. Nonsynonymous divergence was measured from the simulations as the fraction of differences between the human and chimp sequences at first and second codon positions.

We next evaluated whether population genetic models including population size changes, recombination rate variation, and natural selection could generate the correlations that we observed in the empirical datasets. We simulated 100 kb regions consisting of exons, introns, and an intergenic sequence (see Materials and Methods, Figure S5). We examined several different models of selection (see Table S6 for the specific parameter values) and examined the correlation between patterns of genetic variation in the neutrally evolving intergenic sequence and other genomic attributes. Because many studies have found that nonsynonymous mutations are weakly deleterious [22], [26], [28], [84], one model included weak negative selection acting only on nonsynonymous sites (shown in purple in Figure 2). It had been suggested that conserved noncoding sites are also likely to be weakly deleterious [25], [27], [31], so another model included negative selection acting on a fraction of intronic sites (shown in blue in Figure 2). In the third model (shown in orange in Figure 2), most mutations at nonsynonymous positions were negatively selected, but a small fraction was positively selected. Finally, the fourth model added weak negative selection at a fraction of intronic sites to a model where most mutations at nonsynonymous positions were negatively selected, but a small fraction was positively selected.

Our simulations confirmed previous predictions that both hitchhiking and background selection [33], [34], [37]–[41] could generate a positive correlation between genetic diversity at linked neutral sites and recombination rate (Figure 2A and Figure S6A). Importantly, these simulations demonstrated that the background selection effect can occur with weak negative selection acting on many sites simultaneously. Models with negative selection acting on noncoding and coding mutations, as well as models with positive selection, could generate positive correlations similar to those in the observed data (red lines in Figure 2A and Figure S6A).

Models of natural selection predicted a positive correlation between average MAF at linked neutral sites and recombination rate (Figure 2B and Figure S6B). The strongest correlations seen for models with only negative selection were for intermediate strengths of selection (e.g. 25% of intronic sites with *s* = 2.5×10^−4^). Stronger selection (*s* = 5×10^−3^) resulted in a weaker correlation (Table S7). Importantly, models that contained no sites under positive selection predicted a correlation between MAF and recombination rate roughly similar in magnitude to that seen in the observed data (red lines in Figure 2B and Figure S6B). These results suggest that both positive and weak negative selection were capable of affecting allele frequencies at linked neutral sites. Thus, a correlation between allele frequency and recombination rate cannot be taken as unambiguous evidence of positive selection.

In some cases, the correlation coefficients between MAF and recombination rate and diversity and recombination rate were significantly higher than zero under purely neutral models (Figure 2 and Figure S6). We performed coalescent simulations using *ms* [85] under the standard neutral model with different rates of recombination to further investigate this issue. Not only was the variance of the distribution of diversity (or average MAF) greater in simulations without recombination, but the shape of the distribution changed depending on the recombination rate. For example, in the case of a high recombination rate, the distribution of the number of segregating sites approached a Poisson distribution, and was symmetric about its mean. However, with no recombination, the distribution became less symmetric, with a higher mass below the mean and a longer tail to the right (Figure S7). Thus, the median of the distribution of diversity simulated with no recombination was lower than the median of the distribution with the high recombination rate. As such, a weak positive correlation between recombination rate and diversity may be expected. The same arguments hold for understanding the correlation between MAF and recombination rate (Figure S7) and Tajima's *D* and recombination rate (Figure S7, see also [63], [86]). Since we used simulations to interpret the correlations observed in the actual data, this effect did not alter our interpretation.

Previous authors ([70]–[77] and reviewed in [78]) had suggested that a negative correlation between neutral polymorphism and nonsynonymous divergence may be a signature of positive selection that cannot be generated by negative selection and/or demographic processes. In our simulations, a model with negative selection acting on noncoding sites, but where a fraction of coding mutations were positively selected showed a negative correlation between *S_norm_* and *d_N_* (orange points in Figure 2C and Figure S6C). Models that did not include any positive selection, but included negative selection on a fraction of noncoding sites (blue points in Figure 2C and Figure S6C), showed little correlation between these two variables. Thus, for the models investigated here, the negative correlation was specific to models of positive selection. As such, it may offer a way to distinguish between models of negative and positive selection. However, a significant negative correlation was not always seen in models that included some sites under positive selection (green points in Figure 2C and Figure S6C). Instead, the correlation was influenced by the relative amounts of negative versus positive selection. Negative selection made the correlation more positive, while positive selection made the correlation more negative. The correlation ultimately observed was due to the net effect of both types of selection.

**Figure 3:**
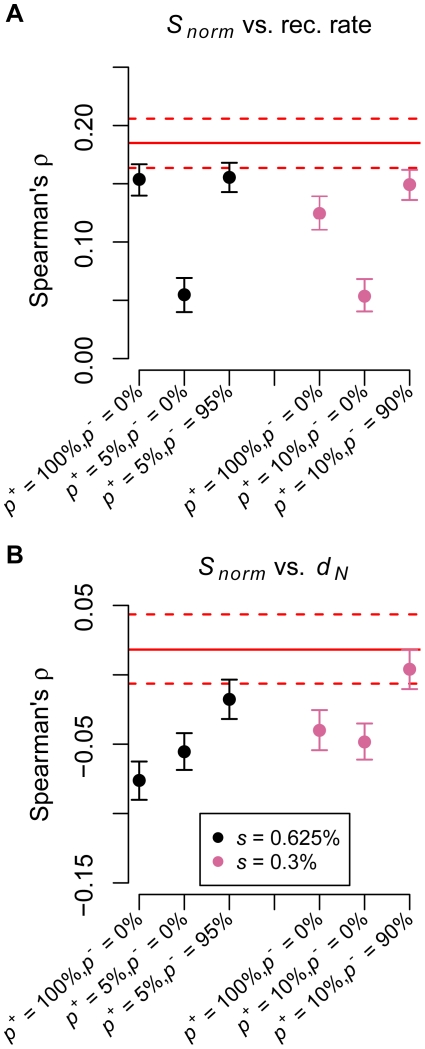
Negative selection is required to match multiple aspects of the low-coverage data. (A) Number of SNPs per covered base divided by human-chimp divergence (*S_norm_*) versus recombination rate. (B) Number of SNPs per covered base divided by human-chimp divergence (*S_norm_*) versus human-chimp nonsynonymous divergence (*d_N_*). The red solid lines denote the point estimates from the genic regions in the low-coverage data. The dotted lines represent 95% confidence intervals obtained by bootstrapping. *p^+^* denotes the proportion of simulated windows that contained positively selected mutations and *p^−^* denotes the proportion of windows that experienced negative selection. All sites in the remaining windows evolved neutrally. In windows with positive selection, 0.5% of nonsynonymous mutations were positively selected (black points: *s* = 0.625%; pink points: *s* = 0.3%), while the remainder evolved neutrally. In windows with negative selection, a gamma distribution of selective effects was used for nonsynonymous mutations and 50% of intronic mutations were selected against with *s* = 0.0075%.

We next used the simulations to evaluate what role positive selection may have played in shaping patterns of variability across the genome. We first examined models with only strong positive selection. A model where 0.5% of nonsynonymous mutations were positively selected (*s* = 0.625%) could generate the observed correlation between *S_norm_* and recombination rate (black, *p^+^* = 100%, *p^−^* = 0% in Figure 3A; *p^+^* denotes the proportion of simulated windows where positive selection could occur). However, this model predicted too strong a negative correlation between *S_norm_* and *d_N_* to be compatible with the data (black, *p^+^* = 100%, *p^−^* = 0% in Figure 3B). Because several studies have suggested that 0–10% of the genome has been affected by a selective sweep [9], [10], [14], [16], [20], [21], we next examined a model where 5% of the simulated windows included positive selection. A model where the remaining 95% of the windows were neutral does not predict a correlation between *S_norm_* and recombination rate strong enough to match the actual data (black, *p^+^* = 5%, *p^−^* = 0% in Figure 3A). This suggests that a small number of positively selected sites by themselves are not sufficient to generate this correlation. Further, this model still predicted a negative correlation between *S_norm_* and *d_N_* (black, *p^+^* = 5%, *p^−^* = 0% in Figure 3B). However, a model where 5% of the simulated windows included positive selection and the remaining 95% of windows included negative selection on coding and noncoding sites predicted a correlation between *S_norm_* and recombination rate similar to that observed in the actual data (black, *p^+^* = 5%, *p^−^* = 95% in Figure 3A). Because adding negative selection resulted in an increase in the strength of this correlation, we concluded that the correlation observed in the data has been primarily driven by negative selection. Also, under this model, the negative correlation between *S_norm_* and *d_N_* was very weak and was compatible with that from the actual data (black, *p^+^* = 5%, *p^−^* = 95% in Figure 3B), presumably because most of the windows have been subjected to negative selection. A model where the strength of positive selection was weaker showed similar trends (pink points in Figure 3). This analysis indicated that the correlation between neutral diversity and recombination rate was primarily driven by many weakly deleterious polymorphisms across the genome, rather than by a small proportion of strongly positively selected mutations.

**Figure 4:**
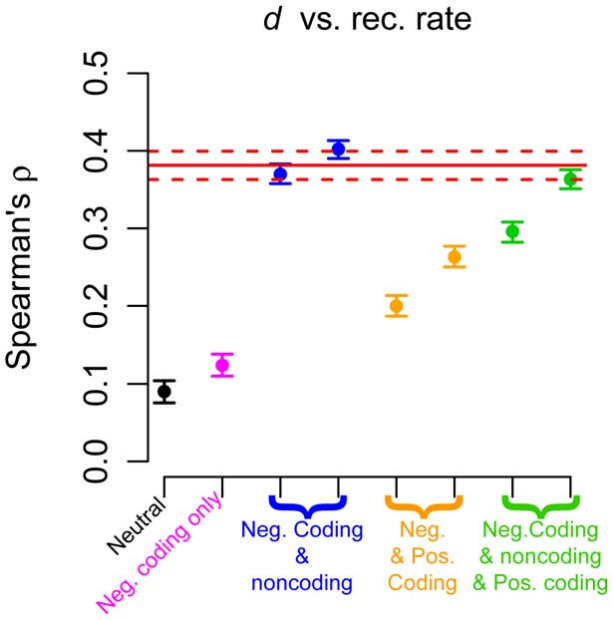
Correlation between neutral human-chimp divergence (*d*) and recombination rate. The red solid line denotes the point estimate from the genic regions in the low-coverage data. The dotted lines represent 95% confidence intervals obtained by bootstrapping. Black points denote a model with no selection and pink points a model where negative selection acted only on nonsynonymous mutations. Blue points denote models where both nonsynonymous and some intronic sites were subjected to negative selection. Orange points denote models where most nonsynonymous mutations were negatively selected, but some were positively selected. Green points denote models where nonsynonymous and some intronic mutations were subjected to negative selection, but a fraction of nonsynonymous mutations were positively selected. See Table S6 for a more detailed description of the different models of selection.

Finally, our simulations (Figure 4) suggest that negative or positive selection can generate a strong correlation between neutral human-chimp divergence (*d*) and recombination rate even when the mutation rate is constant across all simulation replicates. This correlation was likely driven by selection occurring in the ancestral population [67], [87]. Thus, the correlation between *d* and recombination rate can be readily explained by mechanisms other than recombination itself being mutagenic [44], [46], [88].

## Discussion

We have examined patterns of putatively neutral genetic variation in three genome-wide resequencing datasets to gauge the extent of natural selection throughout the human genome. To the best of our knowledge, this is the first report that the allele frequency spectrum is correlated with recombination rate across the human genome (though suggestive evidence was found in smaller datasets [6], [67]). As discussed below, these correlations are best explained by natural selection affecting linked neutral variation across the human genome, rather than artifacts in the data or other mutational processes. Through the use of population genetic simulations, we have shown that a model with negative selection acting on both coding and noncoding mutations fits the data. While we cannot rule out models that include some positive selection, models with abundant positive selection on nonsynonymous mutations and little negative selection predict too strong a negative correlation between neutral polymorphism and nonsynonymous divergence.

In general, we observed qualitatively similar patterns in all three resequencing datasets. However, several of the correlations between different genomic attributes were stronger in the higher-coverage and CGS data than in the low-coverage data (Table 1 and Table 2). Several characteristics of the datasets may contribute to this difference. For example, the higher-coverage and CGS datasets are likely to be of higher quality than the low-coverage dataset. Additionally, a greater proportion of each window is covered in the higher-coverage and CGS datasets than in the low-coverage dataset (Figure S8). Both of these features lead to estimated correlation coefficients that are lower in the low-coverage data than in the higher-coverage and CGS data. Finally, the higher-coverage and CGS datasets contain a sample of a smaller number of chromosomes than the low-coverage data. Population genetic simulations suggest that some of the correlations are expected to be stronger in smaller samples than in larger samples (compare Figure 2 to Figure S6). Thus, the quantitative differences among the correlation coefficients across the different datasets are not too surprising. Instead, the fact that all three datasets show the same general trends is powerful evidence that the correlations are not technical artifacts specific to any one type of data.

Thus, it is our conclusion that these correlations were, at least in part, driven by natural selection across the human genome. Several lines of evidence support this conclusion. First, the correlations remain significant after filtering repetitive sequence and CpG islands (Table S8), and after controlling for the effects of GC content, suggesting that base composition or mutational patterns associated with base composition are not entirely responsible for the correlations.

Second, we have evaluated whether biased gene conversion, a neutral alternative sometimes invoked to explain signatures of natural selection [89], [90], can generate the correlations we have identified. Our simulations show that neutral models with biased gene conversion cannot generate a correlation between *S_norm_* and recombination rate similar in magnitude to that observed in our datasets (Table S6 and Table S7).

The third line of evidence is that the correlation between neutral polymorphism and recombination rate is stronger in genic regions compared to non-genic regions. Natural selection would predominately occur closer to genes, while mutational effects would be distributed throughout the genome [88]. We have also found that both diversity and minor allele frequency are negatively correlated with genic content, suggesting a difference in patterns of variability between genic and non-genic regions of the genome. As discussed further below, models that include natural selection can readily account for these observed patterns.

We have explored which models of selection can generate the correlations that we observed in the actual data. While we have found population genetic models that qualitatively predict the correlations that we have observed in the data, it is more difficult to translate these models into specific statements about the absolute amount of selection in the genome. For example, many of our simulations of negative selection on noncoding sites assume that 25% of intronic sites were under weak negative selection. This is likely to be a substantial over-estimate of the proportion of sites under negative selection [81], [91]. One explanation for this discrepancy is that, for computational convenience, we simulated 100 kb windows independently of each other, rather than whole chromosomes. In reality, each 100 kb window of the genome is linked to other selected mutations outside of the window that may affect patterns of diversity within the window. In fact, simulations of larger windows (348 kb) provide similar values of Spearman's 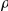 when only 5% of intronic sties are under negative selection (Table S6 and Table S7). This may explain why the models that fit the data include so many selected sites. Simulating larger regions would only yield more biologically relevant simulations if we were able to simulate the correct magnitude of selection at noncoding sites, as well as the correct spatial distribution of sites under selection across the genome. Though there has been some progress from comparative and population genomic studies [25], [27], [31], [81], [91], further work is needed in this area. Additionally, there are nearly an infinite number of possible models for how selection can operate in the genome. For example, selection coefficients within a given window may be correlated with each other, and windows may not be exchangeable (i.e. each window may have its own distribution of selective effects). Our simulations do not capture these phenomena and instead merely illustrate the types of correlations predicted for very basic models of certain types of selection.

Nevertheless, our simplified models do allow some important qualitative statements regarding the relative importance of negative versus positive selection in the human genome. First, all of the correlations observed in all three datasets can be explained without invoking positive selection. Different models of negative selection can readily account for these correlations (Table S6 and Table S7). Second, based on the lack of a negative correlation between *S_norm_* and *d_N_* in any of our datasets (Table S4, Figure 2C, Figure S6C), we can reject models with an abundance of selective sweeps acing on nonsynonymous mutations in the presence of few negatively selected sites (Figure 2, Figure 3, Figure S6). This finding is complementary to what was found in a recent study by Hernandez et al. [21].

However, we cannot rule out the presence of some positively selected mutations in the presence of many negatively selected ones. It is difficult to precisely estimate the fraction of the genome that has been affected by positive selection because such inferences are likely to be highly model-dependent and influenced by many unknown variables. Yet, for the model shown in Figure 3, which fits the actual data (black, *p^+^* = 5%, *p^−^* = 95%), 5% of the simulated windows included positively selected mutations. This model predicts that roughly 2.3% of the windows will have at least one positively selected nonsynonymous mutation that fixed in humans within the last *N_e_* generations (here 20,000 generations, or 500,000 years, assuming 25 years per generation). This is likely to be an upper bound on the fraction of the genome subjected to such strong positive selection because a higher fraction would predict a negative correlation between *S_norm_* and *d_N_* that is too strong to match the data. However, if the strength of positive selection on individual mutations is weaker, if selection operates on standing variation, predominantly on noncoding mutations, or on multiple mutations simultaneously, then a much greater fraction of the genome could have been subjected to positive selection [20], [92]–[94]. Nonetheless, even if a small fraction of the genome was linked to a selective sweep, this amount of selection is not sufficient to generate the correlation between diversity and recombination rate seen in the actual data (Figure 3A). The widespread presence of weakly deleterious alleles, however, can generate this correlation, even in the presence of some positively selected sites (Figure 3A). Taken together, our results suggest that selective sweeps were not the dominant factor explaining the distribution of variability across the human genome.

The notion that sites under natural selection can affect linked neutral variation in the human genome has several important implications for learning about human history using genetic variation data. Most methods to infer parameters in population genetic models assume that all of the SNPs being analyzed are selectively neutral and are not linked to other sites that are affected by selection [17], [95]–[100]. Many of these methods summarize the genetic variation data by the number or proportion of SNPs at different frequencies in the sample (i.e. the frequency spectrum) and then find the demographic parameters that can generate the observed frequency spectrum. Compared to other regions of the genome, we found an excess of low-frequency SNPs in regions near genes and with low recombination rate. It is unlikely that these regions provide an accurate picture of the selectively neutral frequency spectrum for the population of interest. It is unclear what effect including such regions in demographic studies will have on the final parameter estimates. Further investigation of this topic is warranted. In the meantime, one way of circumventing the potential problem of natural selection confounding studies of demography would be to study regions of the genome far away from genes and with high recombination rate [101].

Finally, our study illustrates the utility of low-coverage sequencing data for population genetic studies. Here we have shown that analyzing the low-coverage data without first inferring individual genotypes provides estimates of allele frequency across the genome that are in broad agreement with estimates made from higher-coverage sequencing of a smaller number of individuals. Another unique feature of the low-coverage dataset was that it was generated as part of an exome-capture experiment [84]. Because the capture process is not completely specific and only enriches for sequences within the targeted regions, portions of the genome outside of the targeted regions were sequenced at a lower rate. Such data from a large number of individuals can be used to study patterns of genetic variation across the non-targeted regions of the genome, provided that one analyzes it using an approach that is appropriate for low-coverage data. Such studies promise to yield new insights in population and medical genetics.

## Materials and Methods

### Generation of the low-coverage data

The low-coverage dataset that we used here was an augmented version of the dataset published in Li et al. [84]. The sequencing was performed on 2,000 Danish individuals ascertained from three sources: 1) the population-based Inter99 study [102] (ClinicalTrials.gov ID-no: NCT00289237; *n* = 887), 2) the ADDITION study [103] (ClinicalTrials.gov ID-no: NCT00237548); *n* = 354) and 3) the Steno Diabetes Center (*n* = 759). All participants (mean age of 54.5 years) were of self-reported Danish nationality. All study participants provided written informed consent, and the study was conducted in accordance with the Declaration of Helsinki and approved by the Ethics Committee of Copenhagen County, Denmark. DNA from these individuals was analyzed in an exome-capture resequencing experiment. Each individual was sequenced separately without any pooling. NimbleGen2.1M HD arrays were used to enrich for exome sequences. These arrays contain probes complementary to exonic DNA fragments. Exonic DNA hybridized to the array while non-exonic DNA was washed away. However, this hybridization process was not perfect, and some non-exonic DNA remained bound to the array and was sequenced. The Illumina Genome Analyzer II was used to perform the sequencing. Further methodological details can be found in Li et al. [84].

### Bioinformatic analysis of the low-coverage data

The bioinformatic pipeline used for these data is similar to the one previously published [84]. First, reads were aligned to the NCBI human genome reference assembly (build 36.3) using SOAPaligner [104], [105]. Reads that mapped outside of the exome target regions were retained for further analyses, but bases with a *Q* score <20 were removed. Ideally, since we wish to compare allele frequency estimates for different regions of the genome, we would like to have a similar depth of coverage across the genome. However, depth of coverage varied greatly across the genome with the target regions having very high coverage and the non-target regions having substantially lower coverage. To circumvent this problem, at each position in the genome, we selected a random subset of 100 reads (from the 2,000 individuals) to be used for the frequency estimation process. We chose a cutoff of 100 reads since about 35–40% of the total genome was covered by at least 100 *Q* >20 bases. Decreasing this cutoff would increase the number of bases that were covered, but it would also make it harder to accurately estimate the frequency of lower-frequency SNPs.

We estimated allele frequencies directly from the read counts without attempting to call SNPs or individual genotypes from these data. For each site in the genome with at least 100 reads, we first estimated the population minor allele frequency (MAF) using the method-of-moments estimator 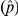 [84]. For sites that had an estimated MAF >1% using 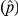, we obtained a more precise estimate of the MAF using the maximum likelihood approach described by Kim et al. [79], [106]. Due to computational constraints on analyzing a dataset of this size, we did not use the genotype likelihood files from soapSNP [107]. Rather, we used the binomial distribution to compute the probability of the read counts for each individual, taking the base-specific sequencing error probabilities into account. We treated the second-most common base at each site as the minor allele. Finally, only sites with estimated MAF >5% were considered as SNPs and were used in subsequent analyses. Given the low depth of coverage (100 reads), it would be difficult to distinguish lower-frequency SNPs from sequencing errors. For example, for a SNP with a MAF of 1%, the less common allele would only be seen approximately one time across all individuals.

### Bioinformatic analysis of the higher-coverage data

We also analyzed a dataset of six European individuals whose genomes were sequenced to higher coverage. This dataset is complementary to the low-coverage dataset because each individual in this dataset was sequenced to higher coverage, coverage was more uniform across the genome, and a higher fraction of bases were covered. But, the sample depth at any particular site in the genome was substantially lower (only 12 chromosomes at most). This dataset included the genomes of James Watson [108], Craig Venter [109], the two parents from a CEU trio (NA12891 and NA12892) that was sequenced to high coverage in pilot 2 of the 1000 Genomes project [69], and two European genomes (NA07022 and NA20431) sequenced by Complete Genomics [110]. Since each individual's genome was sequenced to higher coverage, we treated the called genotypes as though they were the true genotypes throughout subsequent analyses.

For the Venter and Watson genomes, we downloaded SNP genotypes from the “Genome Variants” table of the UCSC browser. Coverage information across these two genomes was obtained from “emf” files from the Ensembl database. Sites with a score of 1 or greater were considered covered. SNPs overlapping regions with a lower score as well as indels and other structural variants were dropped from the analysis. Sites that were covered by reads, but did not have a SNP genotype were considered to be homozygous for the reference genotype.

We downloaded the “.vcf” and “mask” files for the CEU trio of the 1000 Genomes Project. Genotypes for variable positions were obtained from the .vcf files. For the rest of the genome, the individuals were assumed to be homozygous for the reference allele if SNP calling was attempted at the position (i.e. the position had a score of “0” in the mask file). A small number of reported SNPs in the .vcf files that fell in masked positions of the genome were removed from subsequent analyses.

Coverage and SNP genotype information could be directly obtained from the Complete Genomics “variations” files. SNPs and positions that were within 2 bp of indels or structural variants were removed from subsequent analyses.

We intersected the variant genotypes and coverage information from all six genomes and called genotypes for each individual. SNPs with more than two different alleles across all individuals or SNPs where one of the two alleles did not match the reference sequence were removed from subsequent analyses. For sites where one individual had a variant genotype, the genotypes for the other individuals who did not have a variant allele were considered to be homozygous for the reference if they had coverage at that particular site, or were considered to be missing if they did not have any coverage. Subsequent analyses of diversity levels and MAF only used those SNPs and sites that were covered in all six individuals.

### Bioinformatic analysis of the Complete Genomics (CGS) data

We also analyzed six European genomes sequenced by Complete Genomics (CGS). Five of the genomes were from the CEU sample (NA06985, NA06994, NA07357, NA10851, and NA12004) and one was from a TSI individual (NA20502). We used the genotype calls made by CGS that were found in the “masterVarBeta” files. SNPs with more than two different alleles across all individuals, SNPs where one of the two alleles did not match the reference sequence, and sites that were within 2 bp of structural variants called in any one of the individuals were removed from subsequent analyses. Later analyses of diversity levels and MAF only used those SNPs and sites that were covered in all six individuals.

We noted that some windows of the genome appeared to have an unusually high number of SNPs where many individuals were heterozygous (Figure S9). We removed windows which had at least 10 SNPs where the average number of heterozygous genotypes per SNP was greater than 3 (out of 6). This filtering resulted in dropping 3.8% of the windows and appeared to remove the outlier regions (Figure S9).

### Correlation analyses

We divided the genome into non-overlapping 100 kb windows. Windows that were within 10 Mb of an annotated centromere, telomere, or end of a chromosome were omitted from further analyses. For each window, we tabulated several genomic features. First, we obtained the recombination rate for each window using the high-resolution pedigree-based genetic map assembled by deCODE [80]. Second, we tabulated the number of sites within each window where the hg18 base differed from the pantro2 base. This was done using the .axt alignments obtained from the UCSC browser. Importantly, bases in RepeatMasked parts of the genome or where the hg18 or pantro2 alleles were missing were not counted. Since we wanted to examine putatively neutral sites, bases falling in the 17-way phastCons regions were also not counted [81], except when analyzing synonymous and nonsynonymous human-chimp divergence (see below). Third, we tabulated GC content within each window as the fraction of bases where the hg18 sequence was a G or a C. Only those bases that met the inclusion criteria described above were counted in this analysis. Fourth, as a measure of genic content, we tabulated the proportion of bases within each window that overlapped with a RefSeq transcript. We then tabulated the number of SNPs within each window and the number of bases that had sequencing coverage (see above for the criteria used to define covered bases). Importantly, SNPs falling RepeatMasked regions or phastCons regions were dropped from the analysis. Similarly, these bases were not counted as covered bases. The number of SNPs per covered base was used as a summary of diversity within each window. Finally, we summarized the frequency spectrum within each window by the average MAF over all the SNPs within each window.

We tested for correlations between the variables described above using non-parametric correlation tests. Specifically, we tested for pairwise correlations between variables using Spearman's 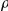. Since many of the variables were correlated with each other (Table S1), we calculated partial correlations to remove the effects of confounding variables on the variables of interest. Partial correlation statistics were calculated using the pcor function in R [111].

We tested whether the correlations were stronger in genic windows compared to non-genic windows using a permutation test. For each permutation, windows were randomly assigned to a genic and a non-genic group, keeping the number of genic and non-genic windows equal to that in the observed data. We recorded the difference in the correlation coefficient between each permuted genic and permuted non-genic dataset. The *P*-value for the test was the proportion of 10,000 permuted datasets with differences larger than those seen in the non-permuted data.

To test for a correlation between neutral polymorphism (*S_norm_*) and nonsynonymous divergence, we found the number of nonsynonymous hg18-pantro2 alignment differences in each window (*D_N_*). This was done by putting those alignment differences that were not in RepeatMasked sequence and overlapped with an exon in the Consensus Coding Sequence (CCDS) table from the UCSC Table Browser into the SeattleSeq SNP annotation pipeline (http://gvs.gs.washington.edu/SeattleSeqAnnotation/). The human and the chimp bases were used as the two alleles. If multiple CCDS genes overlapped, we selected the longest one and discarded the remainder. We used the Nei-Gobjori [112] approach with the CCDS gene model to count the number of synonymous (*L_S_*) and nonsynonymous (*L_N_*) sites per window. *L_N_* and *L_S_* were only counted from the hg18 sequence, rather than averaged between the hg18 and pantro2 sequences. Only those sites that were not Repeat-Masked and were aligned with pantro2 were counted. The number of nonsynonymous differences per nonsynonymous site (*d_N_*) was then calculated as *D_N_*/*L_N_*. Similarly, the number of synonymous differences per synonymous site (*d_S_*) was then calculated as *D_S_*/*L_S_*.

### Simulations

To determine which models of selection could generate the correlations we observed in the resequencing data, we performed forward-in-time population genetic simulations using the program SFS_CODE [113]. Specifically, we simulated 100 kb regions that included exons and introns separated by an intergenic spacer region (Figure S5). We assumed a Jukes-Cantor mutation model [114] with a per-base pair mutation rate of 2.5×10^−8^.

Figure S10 shows the demographic model used for the simulations. Briefly, we simulated a human population with a chimp outgroup where the chimp population split from the human population 5 million years ago (assuming 25 years per generation). The ancestral human-chimp population was assumed to be of size 20,000 because previous studies have found that the ancestral human-chimp population was likely 2–10-fold larger than the current human effective population size [115]–[119]. At the human-chimp speciation event, the both the chimp and human populations underwent an instantaneous 2-fold contraction to their current sizes. Since our data consisted of European individuals, we also included a bottleneck in the human population with parameters from Lohmueller et al. [120], but using an ancestral population size of 10,000 between the human-chimp split and the more recent bottleneck.

The recombination rates for the simulated windows were chosen to approximately match the distribution of estimated recombination rates of the genic windows from the low-coverage dataset. This was done by assigning each window in the low-coverage data to one of 100 different bins based on its recombination rate. A single recombination rate was chosen for each bin (the mid-point of the bin), and this rate was used to simulate the number of replicates proportional to the number of windows in the actual data falling into the bin. A total of 20,000 simulated windows were generated for each model of selection.

Recombination hotspots were added to each window. Hotspots were assumed to have a width of 2 kb and the inter-hotspot distances were drawn from an exponential distribution with a mean of 20 kb. To specify the intensities of the hotspots in SFS_CODE, one needs to provide the proportion of the total amount of recombination that occurred within each of the hotspots and coldspots. We set the proportion of recombination that occurred in hotspot *i* to be 0.8*x_i_*, where *x_i_* was drawn from a Dirichlet distribution with parameter *k* equal to the number of hotspots within the window, and 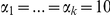. This framework allowed hotspots to have different intensities and kept the total proportion of recombination that occurred in hotspots in each window at 80% [121]. A similar approach was used to determine the background recombination rates for each part of the sequence outside of the hotspots, except 0.2 was used instead of 0.8.

We examined several different models of natural selection (Table S6). In most models, nonsynonymous mutations were weakly deleterious with their selection coefficients drawn from a gamma distribution of selective effects, the parameters of which had been estimated from human resequencing data [22]. Some models also included positive selection acting on a fraction of nonsynonymous mutations, or a fraction of intronic mutations that were weakly deleterious.

We then tabulated diversity and divergence summary statistics from the simulations. Importantly, we only analyzed SNPs and human-chimp differences that occurred in the neutral intergenic sequence. For comparison to the low-coverage Danish data, we used the population MAFs from the simulations, counting only those SNPs with MAF >5% as we did in the observed data. From these same simulations, we took a sample of six individuals to analyze and compare to the higher-coverage data. The strength of some correlations may depend on how precisely diversity statistics could be estimated, and these estimates likely depend on the amount of sequence analyzed within each window (or, in other words, the fraction of bases within the 100 kb window that were covered). Therefore, we sampled the amount of intergenic sequence to be analyzed in each simulated window from the empirical distribution of the number of bases covered in each window. This was done separately for the low and higher-coverage datasets because the number of bases covered differed between the two datasets. To compute the number of human-chimp differences from the simulations, we compared the sequence of a single chimp individual to a single human individual. Sites where the two individuals were homozygous for different alleles were counted as differences. Sites where both were homozygous for the same allele were not counted as differences. All other sites (e.g. chimp was heterozygous and human was homozygous, chimp was heterozygous and human was heterozygous, chimp was homozygous and human was heterozygous) were counted as half a difference.

For computational efficiency, we simulated an ancestral population of 500 individuals while keeping the population-scaled mutation and recombination rates and selection coefficients equal to their original values. This approach increased computational efficiency, but should result in the same patterns of variation as larger population sizes since the patterns of variation depend only on the scaled population parameters.

## Supporting Information

Figure S1Correlations between the number of SNPs per covered base among the three different datasets. The red line denotes the lowess curve fit to the two variables. The value of Spearman's 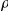 for each pairwise correlation is shown in each panel. Note that several outlier data points fell outside the plotting area.(TIFF)

Figure S2Correlations between the average MAF among the three different datasets. The red line denotes the lowess curve fit to the two variables. The value of Spearman's 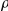 for each pairwise correlation is shown in each panel. Note that several outlier data points fell outside the plotting area.(TIFF)

Figure S3 Correlations between summaries of genetic variation and recombination rate in the higher-coverage dataset dividing the data into genic and non-genic windows (see text). (A) Number of SNPs per covered base divided by human-chimp divergence (*S_norm_*) versus recombination rate. (B) Average minor allele frequency versus recombination rate. Red and green lines denote the lowess curves fit to the two variables for genic and non-genic windows, respectively. Black points denote genic windows while gray points denote non-genic windows. Each point represents the average statistics computed over 50 100 kb windows. The windows were sorted by recombination rate prior to binning. Note that several outlier data points fell outside the plotting area.
(TIFF)

Figure S4 Correlations between summaries of genetic variation and recombination rate in the CGS dataset dividing the data into genic and non-genic windows (see text). (A) Number of SNPs per covered base divided by human-chimp divergence (*S_norm_*) versus recombination rate. (B) Average minor allele frequency versus recombination rate. Red and green lines denote the lowess curves fit to the two variables for genic and non-genic windows, respectively. Black points denote genic windows while gray points denote non-genic windows. Each point represents the average statistics computed over 50 100 kb windows. The windows were sorted by recombination rate prior to binning. Note that several outlier data points fell outside the plotting area.
(TIFF)

Figure S5 Structure of a simulated window. Each window contains 8 exons, 7 introns, and a 53 kb neutral intergenic sequence in the middle. Some models of selection included negative selection only on coding sites. Other models included negative and positive selection on coding sites. A third set of models added negative selection on a fraction of intronic sites. See Table S6 for a further description of the different models of selection.
(TIFF)

Figure S6Comparison of Spearman's 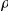 for genic regions with the expected values based on forward simulations for the higher-coverage dataset. (A) Number of SNPs per covered base divided by human-chimp divergence (*S_norm_*) versus recombination rate. (B) Average minor allele frequency versus recombination rate. (C) Number of SNPs per covered base divided by human-chimp divergence (*S_norm_*) versus human-chimp nonsynonymous divergence (*d_N_*). The red solid lines denote the point estimate from the genic regions in the higher-coverage data. The dotted lines represent 95% confidence intervals obtained by bootstrapping. Black points denote a model with no selection and pink points a model where negative selection acted only on nonsynonymous mutations. Blue points denote models where both nonsynonymous and some intronic sites were subjected to negative selection. Orange points denote models where most nonsynonymous mutations were negatively selected, but some were positively selected. Green points denote models where nonsynonymous and some intronic mutations were subjected to negative selection, but a fraction of nonsynonymous mutations were positively selected. See Table S6 for a more detailed description of the different models of selection. Nonsynonymous divergence was measured from the simulations as the fraction of differences between the human and chimp sequences at first and second codon positions.(TIFF)

Figure S7Effect of recombination on the distributions of summaries of neutral genetic variation. (A) Number of SNPs per window. (B) Average number of pairwise differences. (C) Tajima's *D*. (D) Average minor allele frequency. Each figure shows the distribution of the particular summary statistic for 10^5^ simulated (under the standard neutral model using *ms* [85]) 100 kb windows in a sample size of 200 chromosomes assuming no recombination (red curves) and a recombination rate of 10 cM/Mb (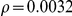 per base pair, black curves). Solid vertical lines denote the medians of the distributions. Dashed vertical lines denote the means of the distributions. In panels A and B, the means of all distributions match the medians of the 10 cM/Mb (black) distributions. In panels C and D, the means of the 10 cM/Mb (black) distributions match the medians.(TIFF)

Figure S8Correlations between the number of bases covered per window among the three different datasets. The red line denotes the lowess curve fit to the two variables. The value of Spearman's 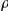 for each pairwise correlation is shown in each panel. Note that several outlier data points fell outside the plotting area.(TIFF)

Figure S9 Patterns of heterozygosity in the CGS data. Number of heterozygous genotypes per window (i.e. the number of heterozygous genotypes per SNP summed over all SNPs within each window) is represented on the y-axis and the number of SNPs per window is represented on the x-axis. Red points denote those windows with at least 10 SNPs where the average number of heterozygous genotypes per SNP was >3 (out of 6). Such windows were excluded from further analyses.
(TIFF)

Figure S10 Demographic model used for simulations. *N_H-C_* denotes the ancestral human-chimp population size, *N_C_* denotes the current chimp population size, *N_H_* denotes the current human population size, *N_HBN_* denotes the human population size during the bottleneck, *t_split_* denotes the human-chimp split time, *t_BN-start_* denotes the time when the population size decreased to start the bottleneck (moving forward in time), and *t_BN-end_* denotes the time when the population recovered from the bottleneck (moving forward in time). Note that all population parameters are scaled by *N_H-C_* = 20,000. However, for computational efficiency, we simulated 500 individuals while keeping the population parameters equal to their original values (see Materials and Methods).
(TIFF)

Table S1 Pairwise correlations between variables for the low-coverage data.
(PDF)

Table S2 Pairwise correlations between variables for the higher-coverage data.
(PDF)

Table S3 Pairwise correlations between variables for the CGS data.
(PDF)

Table S4Correlation coefficients (Spearman's 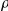) between coding region divergence and neutral diversity (*S_norm_*).(PDF)

Table S5Correlation coefficients (Spearman's 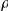) between coding region divergence and neutral diversity (*S_norm_*) for windows in the upper 90^th^ percentile of nonsynonymous divergence per site (*d_N_*) or synonymous divergence per site (*d_S_*).(PDF)

Table S6 Selection models used in forward simulations.
(PDF)

Table S7Values of Spearman's 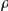 calculated from forward simulations of various models of selection.(PDF)

Table S8 Pairwise correlations between variables for the CGS data after filtering CpG islands.
(PDF)

## References

[1] Akey JM, Zhang G, Zhang K, Jin L, Shriver MD (2002). Interrogating a high-density SNP map for signatures of natural selection.. Genome Res.

[2] Payseur BA, Cutter AD, Nachman MW (2002). Searching for evidence of positive selection in the human genome using patterns of microsatellite variability.. Mol Biol Evol.

[3] Akey JM, Eberle MA, Rieder MJ, Carlson CS, Shriver MD (2004). Population history and natural selection shape patterns of genetic variation in 132 genes.. PLoS Biol.

[4] Storz JF, Payseur BA, Nachman MW (2004). Genome scans of DNA variability in humans reveal evidence for selective sweeps outside of Africa.. Mol Biol Evol.

[5] Carlson CS, Thomas DJ, Eberle MA, Swanson JE, Livingston RJ (2005). Genomic regions exhibiting positive selection identified from dense genotype data.. Genome Res.

[6] Stajich JE, Hahn MW (2005). Disentangling the effects of demography and selection in human history.. Mol Biol Evol.

[7] Kelley JL, Madeoy J, Calhoun JC, Swanson W, Akey JM (2006). Genomic signatures of positive selection in humans and the limits of outlier approaches.. Genome Res.

[8] Voight BF, Kudaravalli S, Wen X, Pritchard JK (2006). A map of recent positive selection in the human genome.. PLoS Biol.

[9] Wang ET, Kodama G, Baldi P, Moyzis RK (2006). Global landscape of recent inferred Darwinian selection for *Homo sapiens*.. Proc Natl Acad Sci U S A.

[10] Hawks J, Wang ET, Cochran GM, Harpending HC, Moyzis RK (2007). Recent acceleration of human adaptive evolution.. Proc Natl Acad Sci U S A.

[11] Nielsen R, Hellmann I, Hubisz M, Bustamante C, Clark AG (2007). Recent and ongoing selection in the human genome.. Nat Rev Genet.

[12] Sabeti PC, Varilly P, Fry B, Lohmueller J, Hostetter E (2007). Genome-wide detection and characterization of positive selection in human populations.. Nature.

[13] Tang K, Thornton KR, Stoneking M (2007). A new approach for using genome scans to detect recent positive selection in the human genome.. PLoS Biol.

[14] Williamson SH, Hubisz MJ, Clark AG, Payseur BA, Bustamante CD (2007). Localizing recent adaptive evolution in the human genome.. PLoS Genet.

[15] Kelley JL, Swanson WJ (2008). Positive selection in the human genome: From genome scans to biological significance.. Annu Rev Genomics Hum Genet.

[16] Akey JM (2009). Constructing genomic maps of positive selection in humans: Where do we go from here?. Genome Res.

[17] Nielsen R, Hubisz MJ, Hellmann I, Torgerson D, Andres AM (2009). Darwinian and demographic forces affecting human protein coding genes.. Genome Res.

[18] Pickrell JK, Coop G, Novembre J, Kudaravalli S, Li JZ (2009). Signals of recent positive selection in a worldwide sample of human populations.. Genome Res.

[19] Grossman SR, Shylakhter I, Karlsson EK, Byrne EH, Morales S (2010). A composite of multiple signals distinguishes causal variants in regions of positive selection.. Science.

[20] Coop G, Pickrell JK, Novembre J, Kudaravalli S, Li J (2009). The role of geography in human adaptation.. PLoS Genet.

[21] Hernandez RD, Kelley JL, Elyashiv E, Melton SC, Auton A (2011). Classic selective sweeps were rare in recent human evolution.. Science.

[22] Boyko AR, Williamson SH, Indap AR, Degenhardt JD, Hernandez RD (2008). Assessing the evolutionary impact of amino acid mutations in the human genome.. PLoS Genet.

[23] Eyre-Walker A, Keightley PD (2009). Estimating the rate of adaptive molecular evolution in the presence of slightly deleterious mutations and population size change.. Mol Biol Evol.

[24] Williamson SH, Hernandez R, Fledel-Alon A, Zhu L, Nielsen R (2005). Simultaneous inference of selection and population growth from patterns of variation in the human genome.. Proc Natl Acad Sci U S A.

[25] Drake JA, Bird C, Nemesh J, Thomas DJ, Newton-Cheh C (2006). Conserved noncoding sequences are selectively constrained and not mutation cold spots.. Nat Genet.

[26] Eyre-Walker A, Woolfit M, Phelps T (2006). The distribution of fitness effects of new deleterious amino acid mutations in humans.. Genetics.

[27] Asthana S, Noble WS, Kryukov G, Grant CE, Sunyaev S (2007). Widely distributed noncoding purifying selection in the human genome.. Proc Natl Acad Sci U S A.

[28] Eyre-Walker A, Keightley PD (2007). The distribution of fitness effects of new mutations.. Nat Rev Genet.

[29] Keightley PD, Eyre-Walker A (2007). Joint inference of the distribution of fitness effects of deleterious mutations and population demography based on nucleotide polymorphism frequencies.. Genetics.

[30] Lohmueller KE, Indap AR, Schmidt S, Boyko AR, Hernandez RD (2008). Proportionally more deleterious genetic variation in European than in African populations.. Nature.

[31] Torgerson DG, Boyko AR, Hernandez RD, Indap A, Hu X (2009). Evolutionary processes acting on candidate *cis*-regulatory regions in humans inferred from patterns of polymorphism and divergence.. PLoS Genet.

[32] Keightley PD, Eyre-Walker A (2010). What can we learn about the distribution of fitness effects of new mutations from DNA sequence data?. Philos Trans R Soc Lond B Biol Sci.

[33] Manyard Smith J, Haigh J (1974). The hitch-hiking effect of a favourable gene.. Genet Res.

[34] Charlesworth B, Morgan MT, Charlesworth D (1993). The effect of deleterious mutations on neutral molecular variation.. Genetics.

[35] Aguade M, Miyashita N, Langley CH (1989). Reduced variation in the *yellow-achaete-scute* region in natural populations of *Drosophila melanogaster*.. Genetics.

[36] Begun DJ, Aquadro CF (1992). Levels of naturally occurring DNA polymorphism correlate with recombination rates in *D. melanogaster*.. Nature.

[37] Kaplan NL, Hudson RR, Langley CH (1989). The “hitchhiking effect” revisited.. Genetics.

[38] Charlesworth D, Charlesworth B, Morgan MT (1995). The pattern of neutral molecular variation under the background selection model.. Genetics.

[39] Hudson RR, Kaplan NL (1995). The coalescent process and background selection.. Philos Trans R Soc Lond B Biol Sci.

[40] Hudson RR, Kaplan NL (1995). Deleterious background selection with recombination.. Genetics.

[41] Nordborg M, Charlesworth B, Charlesworth D (1996). The effect of recombination on background selection.. Genet Res.

[42] Nachman MW, Bauer VL, Crowell SL, Aquadro CF (1998). DNA variability and recombination rates at X-linked loci in humans.. Genetics.

[43] Nachman MW (2001). Single nucleotide polymorphisms and recombination rate in humans.. Trends Genet.

[44] Hellmann I, Ebersberger I, Ptak SE, Paabo S, Przeworski M (2003). A neutral explanation for the correlation of diversity with recombination rates in humans.. Am J Hum Genet.

[45] Payseur BA, Nachman MW (2000). Microsatellite variation and recombination rate in the human genome.. Genetics.

[46] Hellmann I, Prufer K, Ji H, Zody MC, Paabo S (2005). Why do human diversity levels vary at a megabase scale?. Genome Res.

[47] Hellmann I, Mang Y, Gu Z, Li P, de la Vega FM (2008). Population genetic analysis of shotgun assemblies of genomic sequences from multiple individuals.. Genome Res.

[48] Cai JJ, Macpherson JM, Sella G, Petrov DA (2009). Pervasive hitchhiking at coding and regulatory sites in humans.. PLoS Genet.

[49] Tajima F (1989). Statistical method for testing the neutral mutation hypothesis by DNA polymorphism.. Genetics.

[50] Braverman JM, Hudson RR, Kaplan NL, Langley CH, Stephan W (1995). The hitchhiking effect on the site frequency spectrum of DNA polymorphisms.. Genetics.

[51] Simonsen KL, Churchill GA, Aquadro CF (1995). Properties of statistical tests of neutrality for DNA polymorphism data.. Genetics.

[52] Tachida H (2000). Molecular evolution in a multisite nearly neutral mutation model.. J Mol Evol.

[53] Comeron JM, Kreitman M (2002). Population, evolutionary and genomic consequences of interference selection.. Genetics.

[54] Gordo I, Navarro A, Charlesworth B (2002). Muller's ratchet and the pattern of variation at a neutral locus.. Genetics.

[55] Comeron JM, Williford A, Kliman RM (2008). The Hill-Robertson effect: Evolutionary consequences of weak selection and linkage in finite populations.. Heredity.

[56] Kaiser VB, Charlesworth B (2009). The effects of deleterious mutations on evolution in non-recombining genomes.. Trends Genet.

[57] O'Fallon BD, Seger J, Adler FR (2010). A continuous-state coalescent and the impact of weak selection on the structure of gene genealogies.. Mol Biol Evol.

[58] Seger J, Smith WA, Perry JJ, Hunn J, Kaliszewska ZA (2010). Gene genealogies strongly distorted by weakly interfering mutations in constant environments.. Genetics.

[59] Santiago E, Caballero A (1998). Effective size and polymorphism of linked neutral loci in populations under directional selection.. Genetics.

[60] Stephan W, Xing L, Kirby DA, Braverman JM (1998). A test of the background selection hypothesis based on nucleotide data from *Drosophila ananassae*.. Proc Natl Acad Sci U S A.

[61] Langley CH, Lazzaro BP, Phillips W, Heikkinen E, Braverman JM (2000). Linkage disequilibria and the site frequency spectra in the *su(s)* and *su(w^a^)* regions of the *Drosophila melanogaster* X chromosome.. Genetics.

[62] Andolfatto P (2001). Adaptive hitchhiking effects on genome variability.. Curr Opin Genet Dev.

[63] Andolfatto P, Przeworski M (2001). Regions of lower crossing over harbor more rare variants in African populations of *Drosophila melanogaster*.. Genetics.

[64] Payseur BA, Nachman MW (2002). Natural selection at linked sites in humans.. Gene.

[65] Braverman JM, Lazzaro BP, Aguade M, Langley CH (2005). DNA sequence polymorphism and divergence at the *erect wing* and *suppressor of sable* loci of *Drosophila melanogaster* and *D. simulans*.. Genetics.

[66] Stephan W (2010). Genetic hitchhiking versus background selection: The controversy and its implications.. Philos Trans R Soc Lond B Biol Sci.

[67] McVicker G, Gordon D, Davis C, Green P (2009). Widespread genomic signatures of natural selection in hominid evolution.. PLoS Genet.

[68] Payseur BA, Nachman MW (2002). Gene density and human nucleotide polymorphism.. Mol Biol Evol.

[69] Durbin RM, Abecasis GR, Altshuler DL, Auton A, 1000 Genomes Project Consortium (2010). A map of human genome variation from population-scale sequencing.. Nature.

[70] Andolfatto P (2007). Hitchhiking effects of recurrent beneficial amino acid substitutions in the *Drosophila melanogaster* genome.. Genome Res.

[71] Macpherson JM, Sella G, Davis JC, Petrov DA (2007). Genomewide spatial correspondence between nonsynonymous divergence and neutral polymorphism reveals extensive adaptation in Drosophila.. Genetics.

[72] Shapiro JA, Huang W, Zhang C, Hubisz MJ, Lu J (2007). Adaptive genic evolution in the Drosophila genomes.. Proc Natl Acad Sci U S A.

[73] Bachtrog D (2008). Similar rates of protein adaptation in *Drosophila miranda* and *D. melanogaster*, two species with different current effective population sizes.. BMC Evol Biol.

[74] Palme AE, Wright M, Savolainen O (2008). Patterns of divergence among conifer ESTs and polymorphism in *Pinus sylvestris* identify putative selective sweeps.. Mol Biol Evol.

[75] Ingvarsson PK (2010). Natural selection on synonymous and nonsynonymous mutations shapes patterns of polymorphism in *Populus tremula*.. Mol Biol Evol.

[76] Jensen JD, Bachtrog D (2010). Characterizing recurrent positive selection at fast-evolving genes in *Drosophila miranda* and *Drosophila pseudoobscura*.. Genome Biol Evol.

[77] Haddrill PR, Zeng K, Charlesworth B (2011). Determinants of synonymous and nonsynonymous variability in three species of Drosophila.. Mol Biol Evol.

[78] Sella G, Petrov DA, Przeworski M, Andolfatto P (2009). Pervasive natural selection in the Drosophila genome?. PLoS Genet.

[79] Kim SY, Lohmueller KE, Albrechtsen A, Li Y, Korneliussen T (2011). Estimation of allele frequency and association mapping using next-generation sequencing data.. BMC Bioinformatics.

[80] Kong A, Thorleifsson G, Gudbjartsson DF, Masson G, Sigurdsson A (2010). Fine-scale recombination rate differences between sexes, populations and individuals.. Nature.

[81] Siepel A, Bejerano G, Pedersen JS, Hinrichs AS, Hou M (2005). Evolutionarily conserved elements in vertebrate, insect, worm, and yeast genomes.. Genome Res.

[82] Larracuente AM, Sackton TB, Greenberg AJ, Wong A, Singh ND (2008). Evolution of protein-coding genes in Drosophila.. Trends Genet.

[83] Tajima F (1990). Relationship between DNA polymorphism and fixation time.. Genetics.

[84] Li Y, Vinckenbosch N, Tian G, Huerta-Sanchez E, Jiang T (2010). Resequencing of 200 human exomes identifies an excess of low-frequency non-synonymous coding variants.. Nat Genet.

[85] Hudson RR (2002). Generating samples under a Wright-Fisher neutral model of genetic variation.. Bioinformatics.

[86] Thornton K (2005). Recombination and the properties of Tajima's *D* in the context of approximate-likelihood calculation.. Genetics.

[87] Begun DJ, Holloway AK, Stevens K, Hillier LW, Poh YP (2007). Population genomics: Whole-genome analysis of polymorphism and divergence in *Drosophila simulans*.. PLoS Biol.

[88] Lercher MJ, Hurst LD (2002). Human SNP variability and mutation rate are higher in regions of high recombination.. Trends Genet.

[89] Galtier N, Duret L (2007). Adaptation or biased gene conversion? Extending the null hypothesis of molecular evolution.. Trends Genet.

[90] Duret L, Galtier N (2009). Biased gene conversion and the evolution of mammalian genomic landscapes.. Annu Rev Genomics Hum Genet.

[91] Pollard KS, Hubisz MJ, Rosenbloom KR, Siepel A (2010). Detection of nonneutral substitution rates on mammalian phylogenies.. Genome Res.

[92] Hancock AM, Alkorta-Aranburu G, Witonsky DB, Di Rienzo A (2010). Adaptations to new environments in humans: The role of subtle allele frequency shifts.. Philos Trans R Soc Lond B Biol Sci.

[93] Pritchard JK, Di Rienzo A (2010). Adaptation - not by sweeps alone.. Nat Rev Genet.

[94] Pritchard JK, Pickrell JK, Coop G (2010). The genetics of human adaptation: Hard sweeps, soft sweeps, and polygenic adaptation.. Curr Biol.

[95] Adams AM, Hudson RR (2004). Maximum-likelihood estimation of demographic parameters using the frequency spectrum of unlinked single-nucleotide polymorphisms.. Genetics.

[96] Marth GT, Czabarka E, Murvai J, Sherry ST (2004). The allele frequency spectrum in genome-wide human variation data reveals signals of differential demographic history in three large world populations.. Genetics.

[97] Voight BF, Adams AM, Frisse LA, Qian Y, Hudson RR (2005). Interrogating multiple aspects of variation in a full resequencing data set to infer human population size changes.. Proc Natl Acad Sci U S A.

[98] Keinan A, Mullikin JC, Patterson N, Reich D (2007). Measurement of the human allele frequency spectrum demonstrates greater genetic drift in East Asians than in Europeans.. Nat Genet.

[99] Gutenkunst RN, Hernandez RD, Williamson SH, Bustamante CD (2009). Inferring the joint demographic history of multiple populations from multidimensional SNP frequency data.. PLoS Genet.

[100] Wall JD, Lohmueller KE, Plagnol V (2009). Detecting ancient admixture and estimating demographic parameters in multiple human populations.. Mol Biol Evol.

[101] Hammer MF, Woerner AE, Mendez FL, Watkins JC, Cox MP (2010). The ratio of human X chromosome to autosome diversity is positively correlated with genetic distance from genes.. Nat Genet.

[102] Jorgensen T, Borch-Johnsen K, Thomsen TF, Ibsen H, Glumer C (2003). A randomized non-pharmacological intervention study for prevention of ischaemic heart disease: Baseline results Inter99.. Eur J Cardiovasc Prev Rehabil.

[103] Lauritzen T, Griffin S, Borch-Johnsen K, Wareham NJ, Wolffenbuttel BH (2000). The ADDITION study: Proposed trial of the cost-effectiveness of an intensive multifactorial intervention on morbidity and mortality among people with type 2 diabetes detected by screening.. Int J Obes Relat Metab Disord.

[104] Li R, Li Y, Kristiansen K, Wang J (2008). SOAP: Short oligonucleotide alignment program.. Bioinformatics.

[105] Li R, Yu C, Li Y, Lam TW, Yiu SM (2009). SOAP2: An improved ultrafast tool for short read alignment.. Bioinformatics.

[106] Kim SY, Li Y, Guo Y, Li R, Holmkvist J (2010). Design of association studies with pooled or un-pooled next-generation sequencing data.. Genet Epidemiol.

[107] Li R, Li Y, Fang X, Yang H, Wang J (2009). SNP detection for massively parallel whole-genome resequencing.. Genome Res.

[108] Wheeler DA, Srinivasan M, Egholm M, Shen Y, Chen L (2008). The complete genome of an individual by massively parallel DNA sequencing.. Nature.

[109] Levy S, Sutton G, Ng PC, Feuk L, Halpern AL (2007). The diploid genome sequence of an individual human.. PLoS Biol.

[110] Drmanac R, Sparks AB, Callow MJ, Halpern AL, Burns NL (2010). Human genome sequencing using unchained base reads on self-assembling DNA nanoarrays.. Science.

[111] Kim SH, Yi SV (2007). Understanding relationship between sequence and functional evolution in yeast proteins.. Genetica.

[112] Nei M, Gojobori T (1986). Simple methods for estimating the numbers of synonymous and nonsynonymous nucleotide substitutions.. Mol Biol Evol.

[113] Hernandez RD (2008). A flexible forward simulator for populations subject to selection and demography.. Bioinformatics.

[114] Jukes TH, Cantor CR, Munro H (1969). Evolution of protein molecules.. Mammalian protein metabolism.

[115] Takahata N, Satta Y, Klein J (1995). Divergence time and population size in the lineage leading to modern humans.. Theor Popul Biol.

[116] Rannala B, Yang Z (2003). Bayes estimation of species divergence times and ancestral population sizes using DNA sequences from multiple loci.. Genetics.

[117] Wall JD (2003). Estimating ancestral population sizes and divergence times.. Genetics.

[118] Hobolth A, Christensen OF, Mailund T, Schierup MH (2007). Genomic relationships and speciation times of human, chimpanzee, and gorilla inferred from a coalescent hidden Markov model.. PLoS Genet.

[119] Burgess R, Yang Z (2008). Estimation of hominoid ancestral population sizes under Bayesian coalescent models incorporating mutation rate variation and sequencing errors.. Mol Biol Evol.

[120] Lohmueller KE, Bustamante CD, Clark AG (2009). Methods for human demographic inference using haplotype patterns from genomewide single-nucleotide polymorphism data.. Genetics.

[121] Myers S, Bottolo L, Freeman C, McVean G, Donnelly P (2005). A fine-scale map of recombination rates and hotspots across the human genome.. Science.

