# Supplementary figures and images for: Natural Selection Affects Multiple Aspects of Genetic Variation at Putatively Neutral Sites across the Human Genome
Source: PLoS Genet. 2011 Oct 13;7(10):e1002326. doi: 10.1371/journal.pgen.1002326 (PMC3192825; doi:10.1371/journal.pgen.1002326)

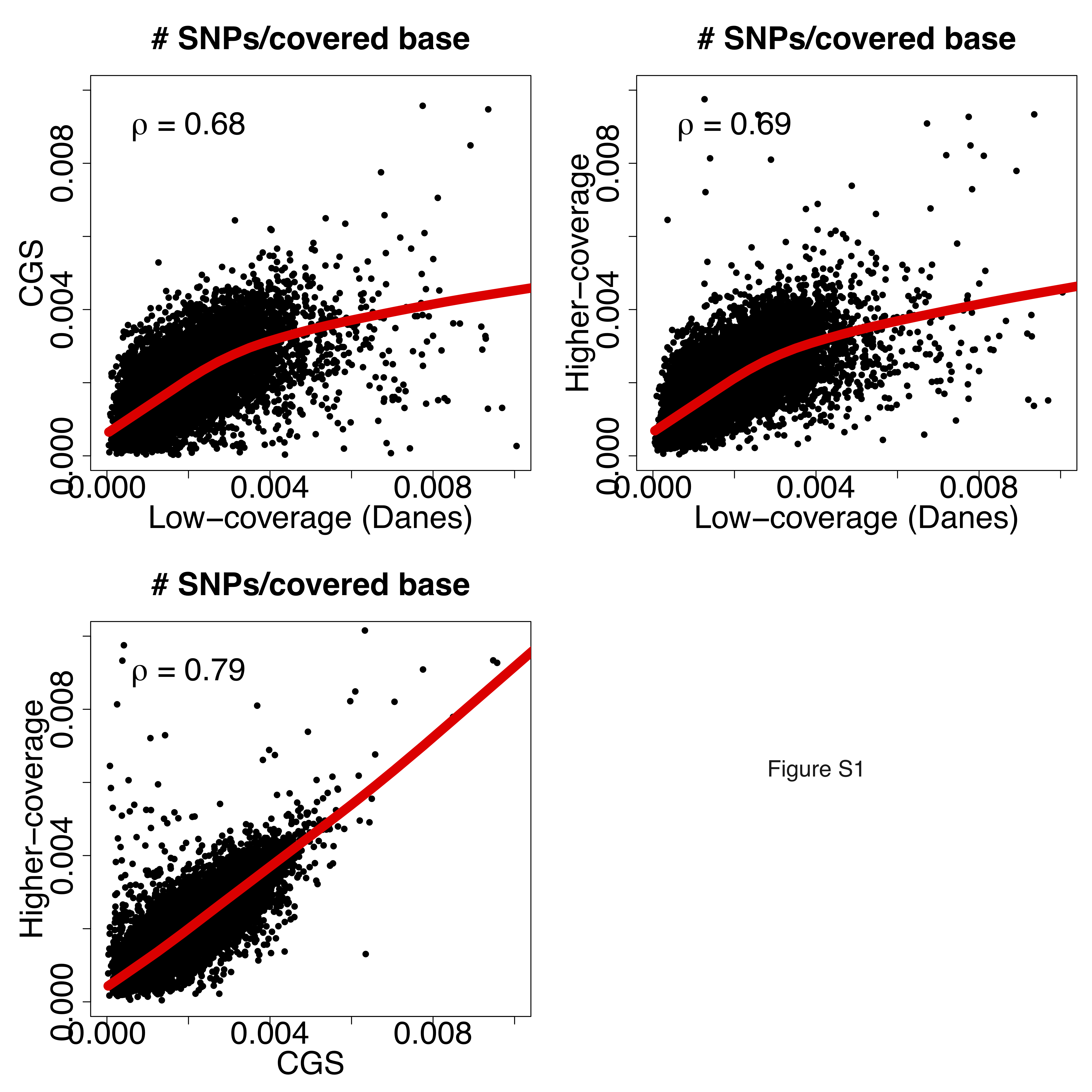

Supplement: Figure S1 — Correlations between the number of SNPs per covered base among the three different datasets. The red line denotes the lowess curve fit to the two variables. The value of Spearman's for each pairwise correlation is shown in each panel. Note that several outlier data points fell outside the plotting area. (TIFF) [file pgen.1002326.s001.tiff]

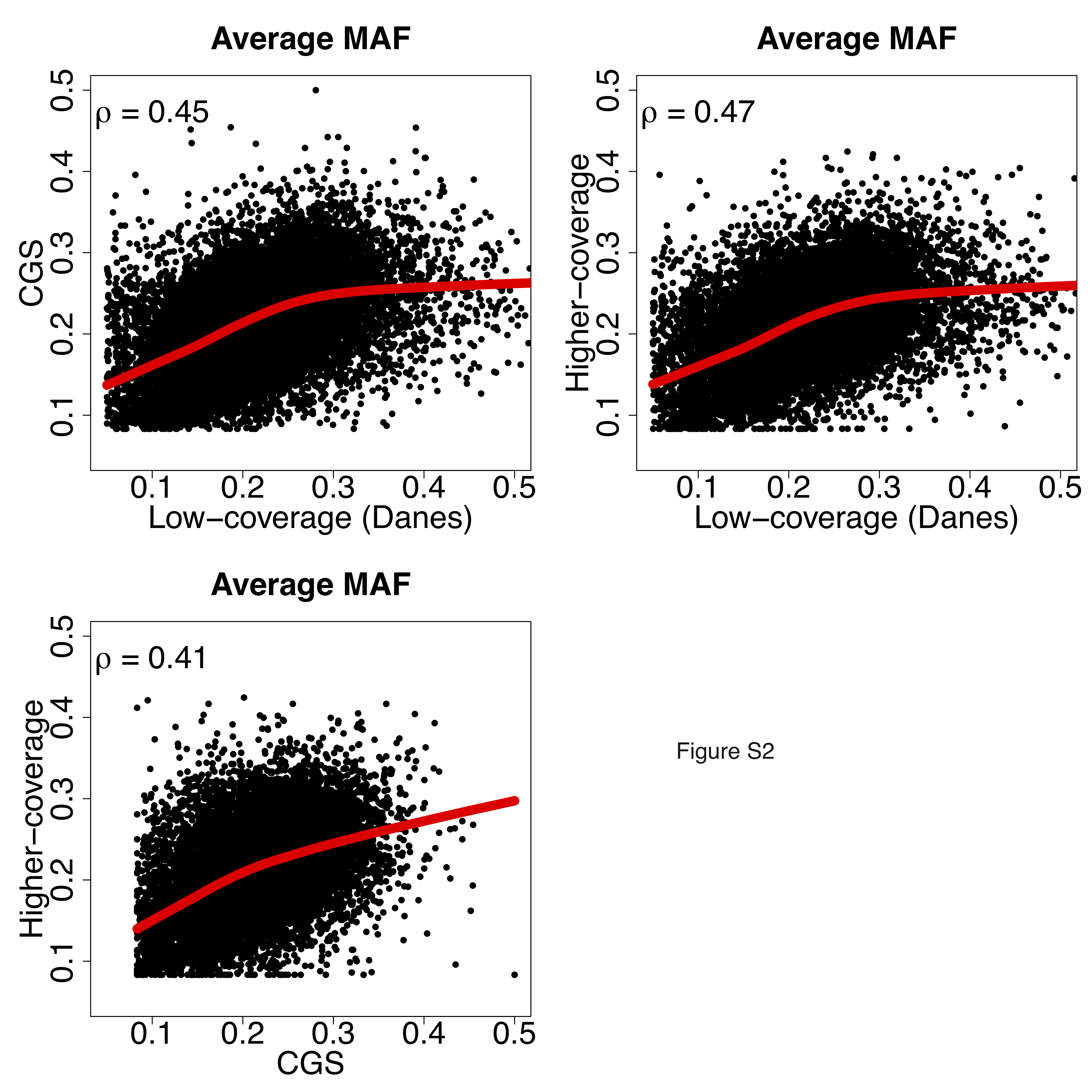

Supplement: Figure S2 — Correlations between the average MAF among the three different datasets. The red line denotes the lowess curve fit to the two variables. The value of Spearman's for each pairwise correlation is shown in each panel. Note that several outlier data points fell outside the plotting area. (TIFF) [file pgen.1002326.s002.tiff]

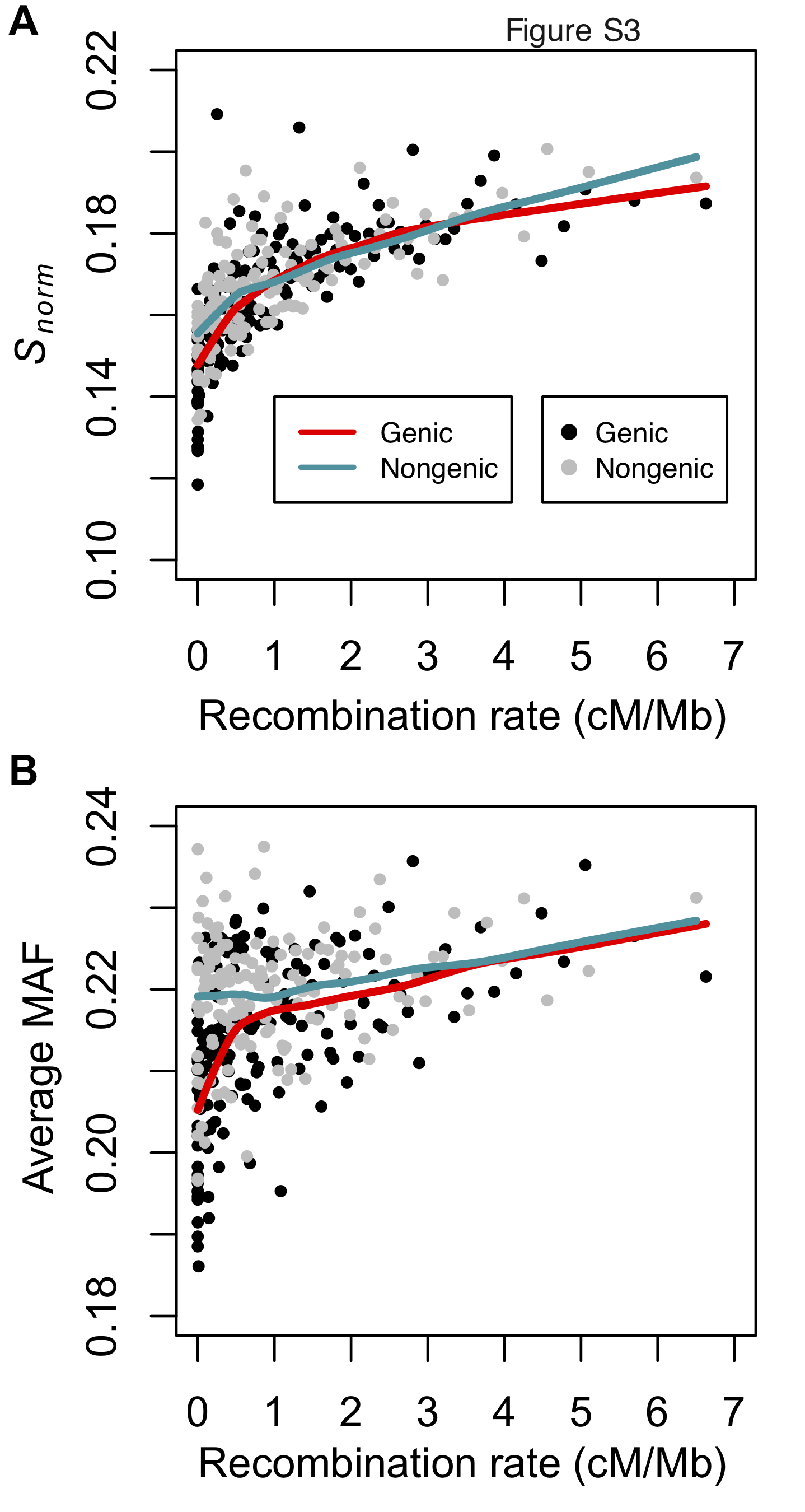

Supplement: Figure S3 — Correlations between summaries of genetic variation and recombination rate in the higher-coverage dataset dividing the data into genic and non-genic windows (see text). (A) Number of SNPs per covered base divided by human-chimp divergence (Snorm) versus recombination rate. (B) Average minor allele frequency versus recombination rate. Red and green lines denote the lowess curves fit to the two variables for genic and non-genic windows, respectively. Black points denote genic windows while gray points denote non-genic windows. Each point represents the average statistics computed over 50 100 kb windows. The windows were sorted by recombination rate prior to binning. Note that several outlier data points fell outside the plotting area. (TIFF) [file pgen.1002326.s003.tiff]

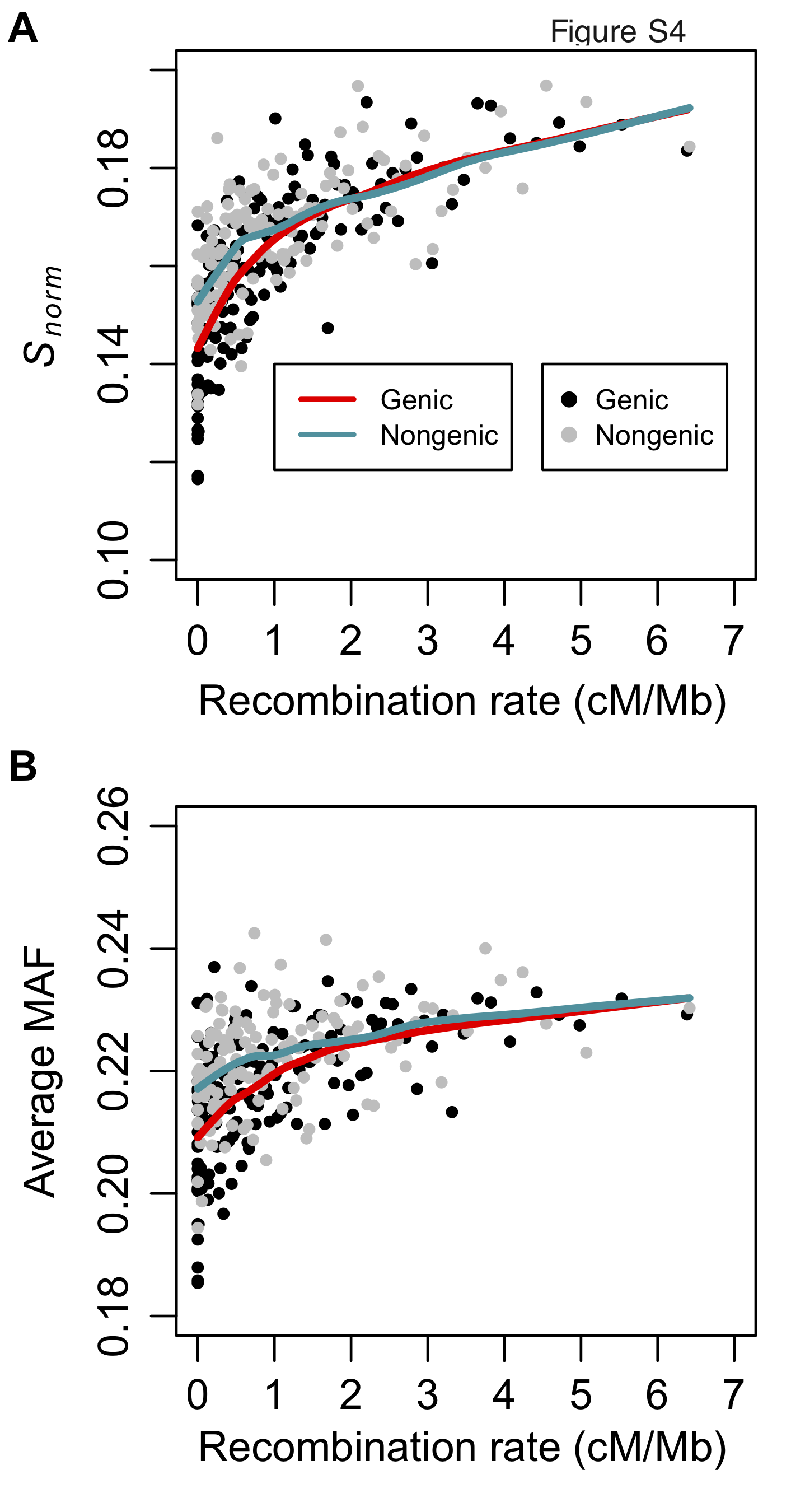

Supplement: Figure S4 — Correlations between summaries of genetic variation and recombination rate in the CGS dataset dividing the data into genic and non-genic windows (see text). (A) Number of SNPs per covered base divided by human-chimp divergence (Snorm) versus recombination rate. (B) Average minor allele frequency versus recombination rate. Red and green lines denote the lowess curves fit to the two variables for genic and non-genic windows, respectively. Black points denote genic windows while gray points denote non-genic windows. Each point represents the average statistics computed over 50 100 kb windows. The windows were sorted by recombination rate prior to binning. Note that several outlier data points fell outside the plotting area. (TIFF) [file pgen.1002326.s004.tiff]

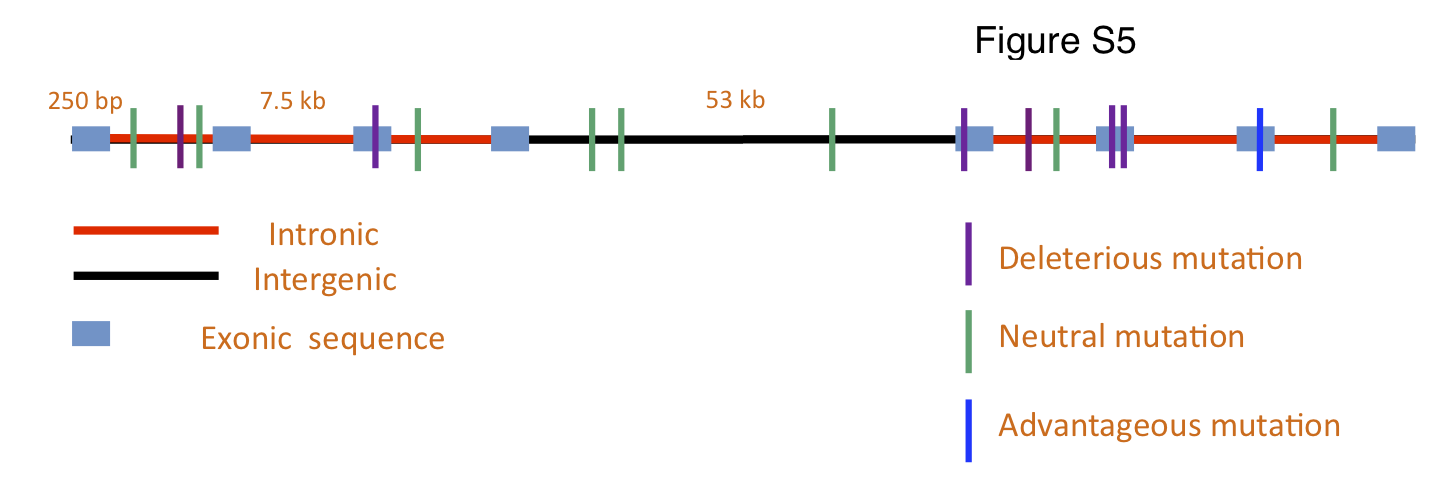

Supplement: Figure S5 — Structure of a simulated window. Each window contains 8 exons, 7 introns, and a 53 kb neutral intergenic sequence in the middle. Some models of selection included negative selection only on coding sites. Other models included negative and positive selection on coding sites. A third set of models added negative selection on a fraction of intronic sites. See Table S6 for a further description of the different models of selection. (TIFF) [file pgen.1002326.s005.tiff]

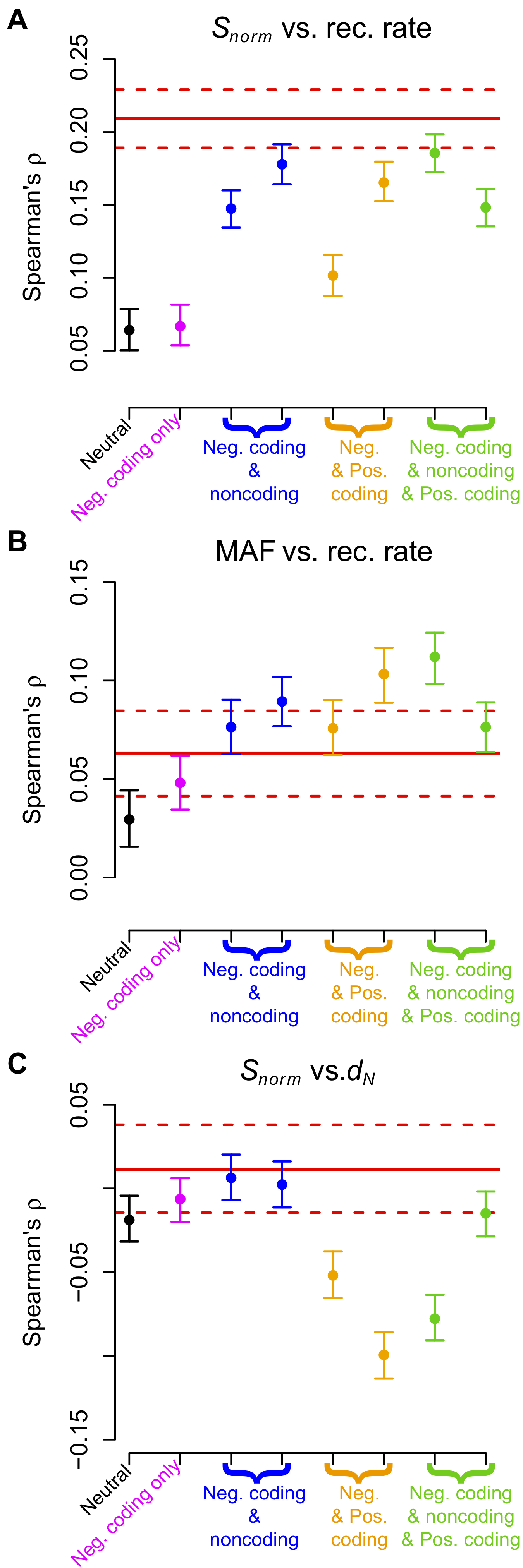

Supplement: Figure S6 — Comparison of Spearman's for genic regions with the expected values based on forward simulations for the higher-coverage dataset. (A) Number of SNPs per covered base divided by human-chimp divergence (Snorm) versus recombination rate. (B) Average minor allele frequency versus recombination rate. (C) Number of SNPs per covered base divided by human-chimp divergence (Snorm) versus human-chimp nonsynonymous divergence (dN). The red solid lines denote the point estimate from the genic regions in the higher-coverage data. The dotted lines represent 95% confidence intervals obtained by bootstrapping. Black points denote a model with no selection and pink points a model where negative selection acted only on nonsynonymous mutations. Blue points denote models where both nonsynonymous and some intronic sites were subjected to negative selection. Orange points denote models where most nonsynonymous mutations were negatively selected, but some were positively selected. Green points denote models where nonsynonymous and some intronic mutations were subjected to negative selection, but a fraction of nonsynonymous mutations were positively selected. See Table S6 for a more detailed description of the different models of selection. Nonsynonymous divergence was measured from the simulations as the fraction of differences between the human and chimp sequences at first and second codon positions. (TIFF) [file pgen.1002326.s006.tiff]

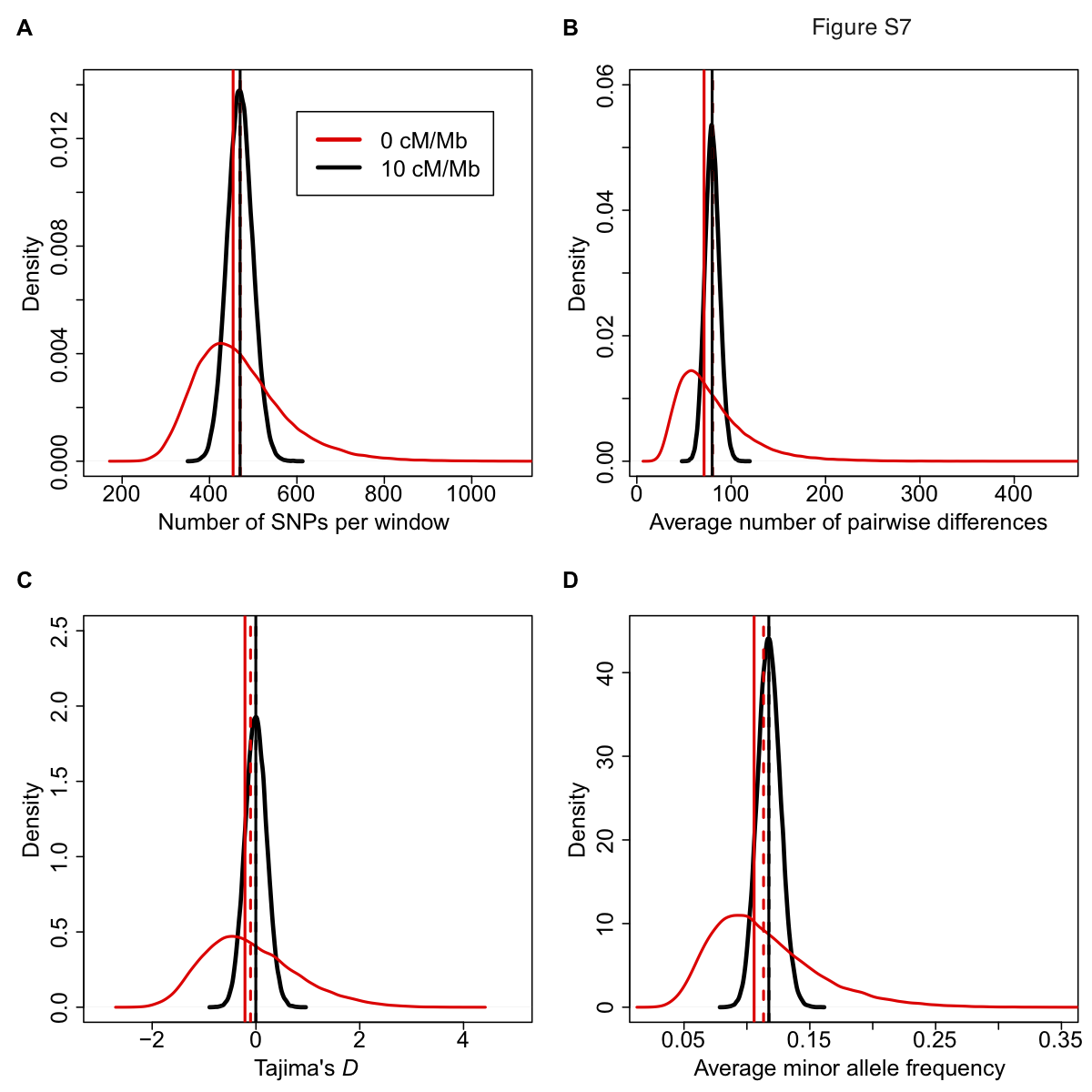

Supplement: Figure S7 — Effect of recombination on the distributions of summaries of neutral genetic variation. (A) Number of SNPs per window. (B) Average number of pairwise differences. (C) Tajima's D. (D) Average minor allele frequency. Each figure shows the distribution of the particular summary statistic for 105 simulated (under the standard neutral model using ms [85]) 100 kb windows in a sample size of 200 chromosomes assuming no recombination (red curves) and a recombination rate of 10 cM/Mb ( per base pair, black curves). Solid vertical lines denote the medians of the distributions. Dashed vertical lines denote the means of the distributions. In panels A and B, the means of all distributions match the medians of the 10 cM/Mb (black) distributions. In panels C and D, the means of the 10 cM/Mb (black) distributions match the medians. (TIFF) [file pgen.1002326.s007.tiff]

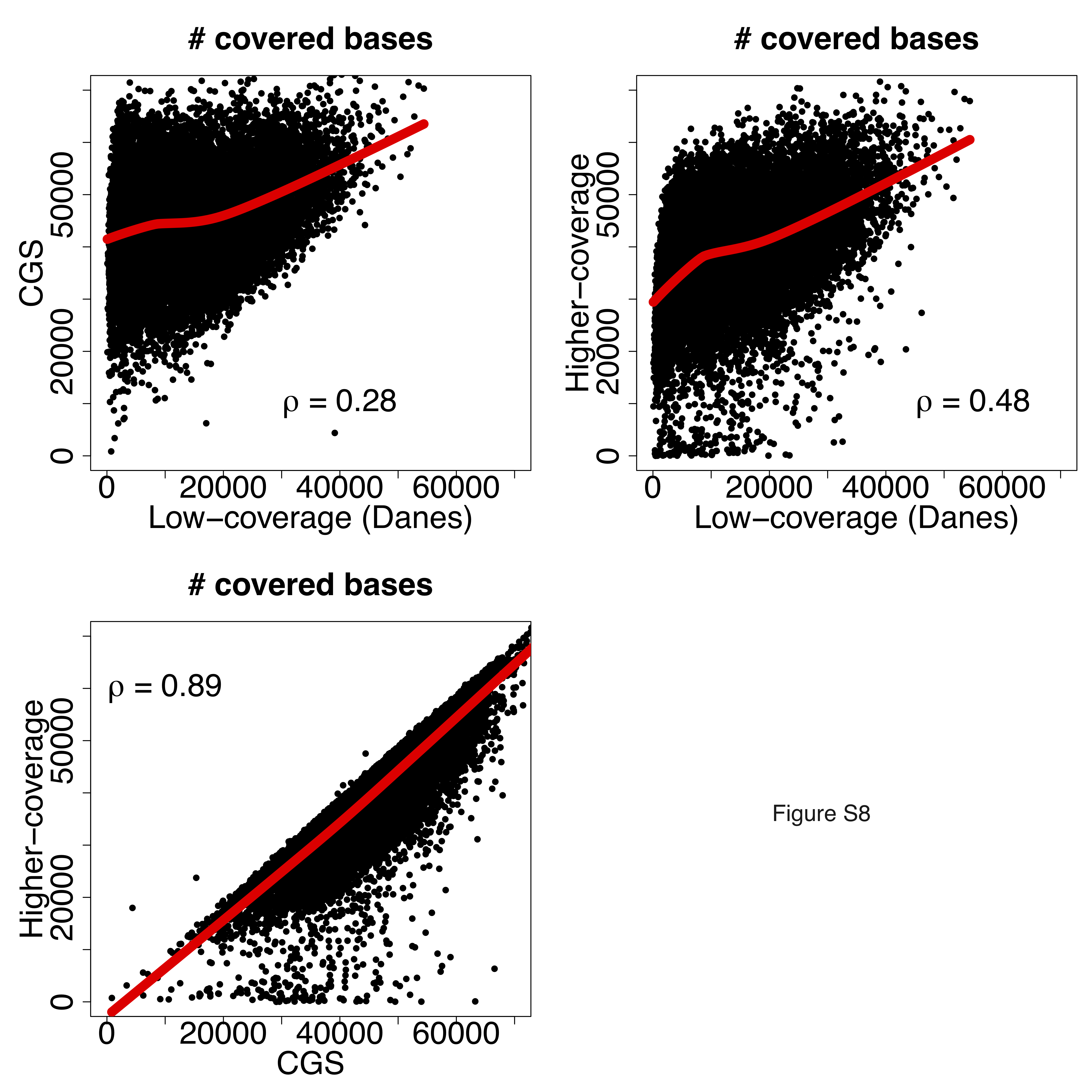

Supplement: Figure S8 — Correlations between the number of bases covered per window among the three different datasets. The red line denotes the lowess curve fit to the two variables. The value of Spearman's for each pairwise correlation is shown in each panel. Note that several outlier data points fell outside the plotting area. (TIFF) [file pgen.1002326.s008.tiff]

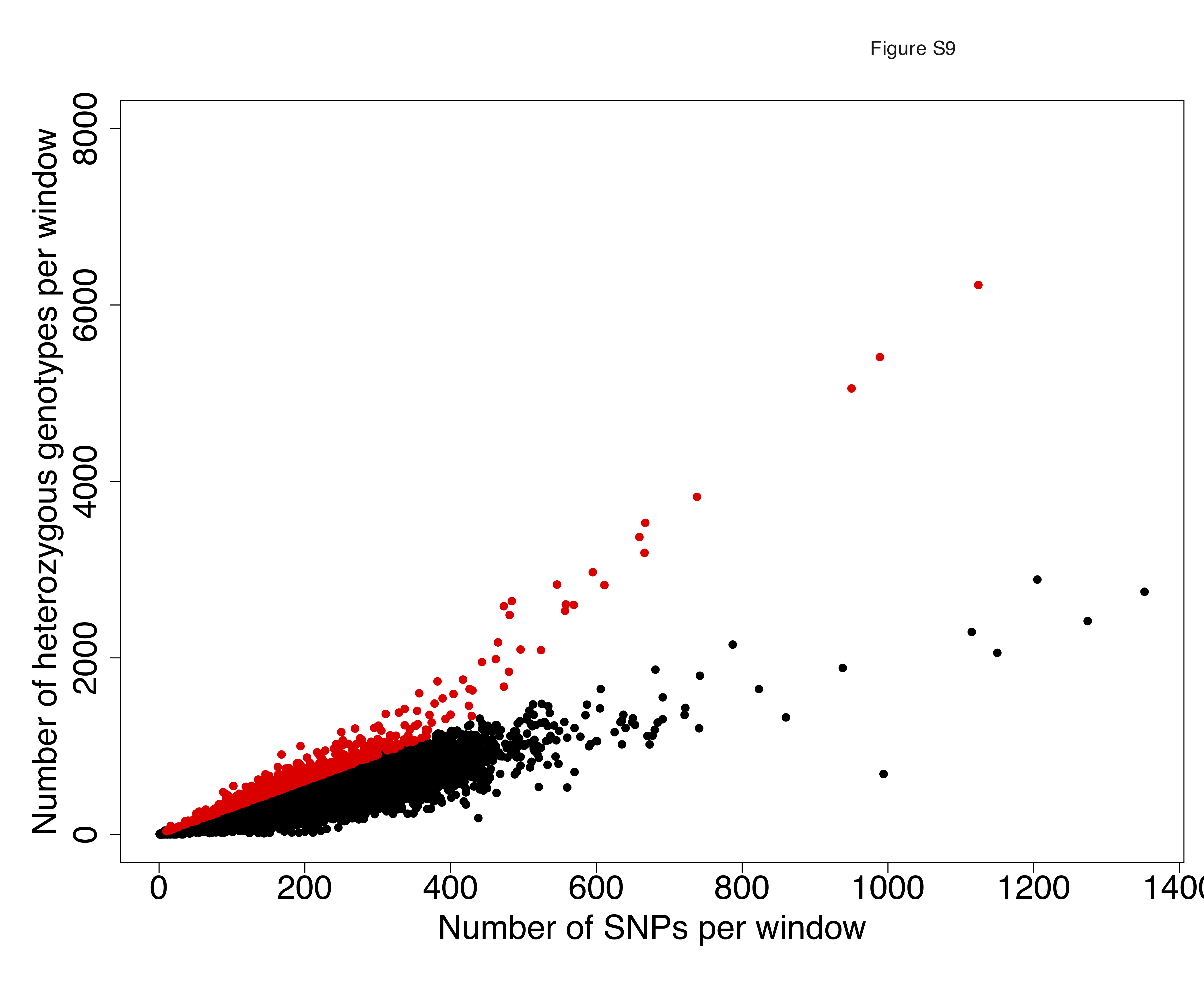

Supplement: Figure S9 — Patterns of heterozygosity in the CGS data. Number of heterozygous genotypes per window (i.e. the number of heterozygous genotypes per SNP summed over all SNPs within each window) is represented on the y-axis and the number of SNPs per window is represented on the x-axis. Red points denote those windows with at least 10 SNPs where the average number of heterozygous genotypes per SNP was >3 (out of 6). Such windows were excluded from further analyses. (TIFF) [file pgen.1002326.s009.tiff]

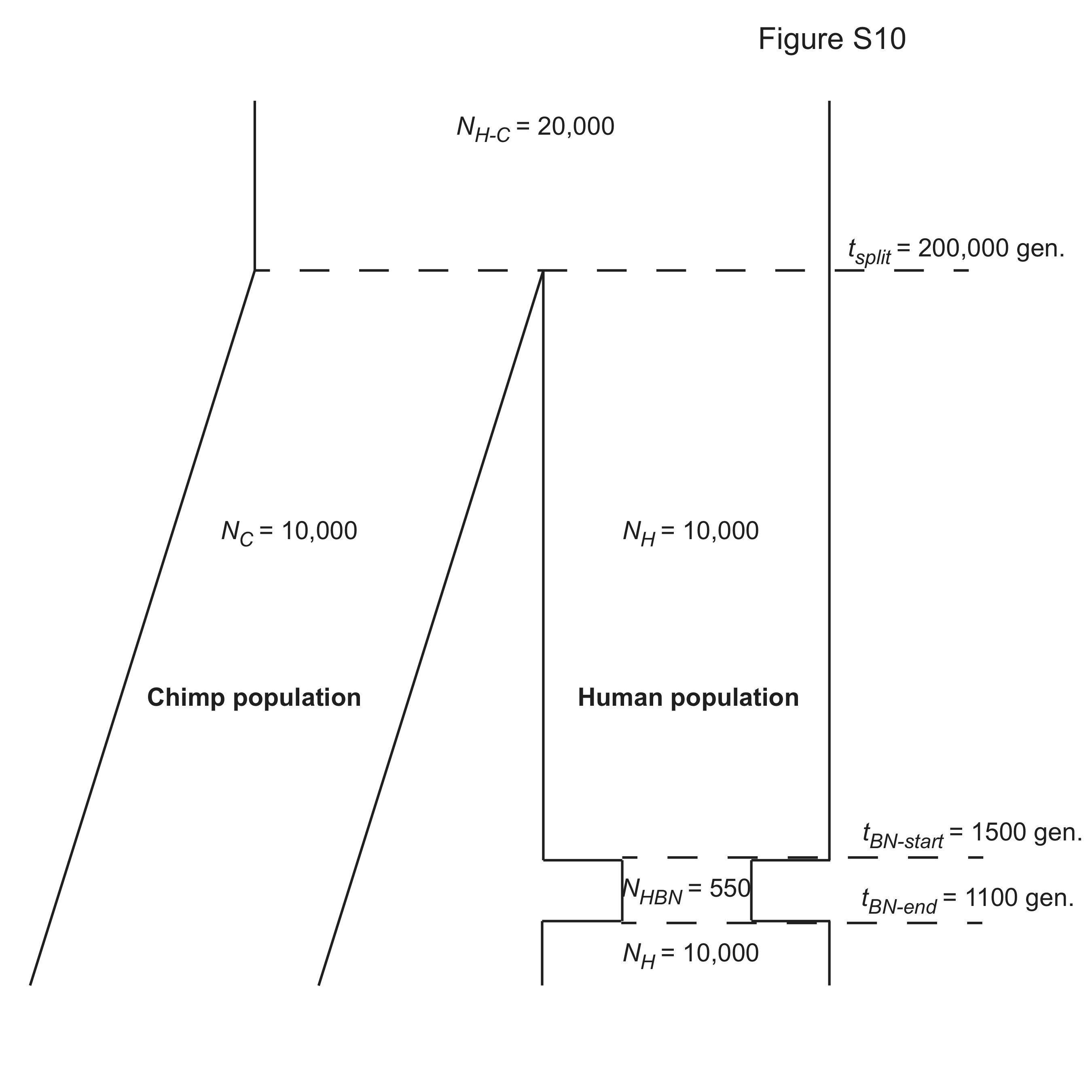

Supplement: Figure S10 — Demographic model used for simulations. NH-C denotes the ancestral human-chimp population size, NC denotes the current chimp population size, NH denotes the current human population size, NHBN denotes the human population size during the bottleneck, tsplit denotes the human-chimp split time, tBN-start denotes the time when the population size decreased to start the bottleneck (moving forward in time), and tBN-end denotes the time when the population recovered from the bottleneck (moving forward in time). Note that all population parameters are scaled by NH-C = 20,000. However, for computational efficiency, we simulated 500 individuals while keeping the population parameters equal to their original values (see Materials and Methods). (TIFF) [file pgen.1002326.s010.tiff]
