# Supplementary material for: Natural Selection Affects Multiple Aspects of Genetic Variation at Putatively Neutral Sites across the Human Genome
Source: PLoS Genet. 2011 Oct 13;7(10):e1002326. doi: 10.1371/journal.pgen.1002326 (PMC3192825; doi:10.1371/journal.pgen.1002326)
Supplement: Table S1 — Pairwise correlations between variables for the low-coverage data. (PDF) [file pgen.1002326.s011.pdf]

Table S1: Pairwise correlations between variables for the low-coverage data.

	Rec. rate	Genic content[a]	GC content[b]	Coverage[c]	d[d]	# SNPs[e]	S_{norm}[f]	Average MAF
Rec. rate		-0.0781	0.3671	0.2662	0.2444	0.1995	0.1113	0.0624
Genic content	4.3E-27		0.1307	0.3869	-0.3074	-0.1456	-0.0388	-0.0120
GC content	0.0E+00	3.9E-73		0.8072	-0.0168	0.0405	0.0271	0.0423
Coverage	1.1E-305	0.0E+00	0.0E+00		-0.1754	-0.0207	0.0320	0.0422
Divergence	1.7E-256	0.0E+00	2.1E-02	42E-131		0.3437	-0.0315	0.0308
# SNPs	7.9E-170	1.4E-90	2.4E-08	4.4E-03	0		0.9037	0.0869
S_{norm}	1.9E-53	8.6E-08	1.8E-04	1.0E-05	1.4E-05	0		0.0806
Average MAF	7.1E-18	9.9E-02	5.3E-09	6.0E-09	2.1E-05	3.7E-33	0	

Values of Spearman's ρ for each pair of variables are shown above the diagonal. P-values are shown below the diagonal.

[a.] Denotes the fraction of each 100 kb window that overlapped with a RefSeq transcript.
[b.] Denotes the number of hg18-pantro2 alignable bases that were not Repeat Masked and did not fall in phastCons regions that were G or C in hg18 divided by the total number of alignable bases within the window that were not Repeat Masked and did not fall in phastCons regions.
[c.] Denotes the number of bases per window that were alignable between hg18 and pantro2, were not Repeat Masked, did not fall in phastCons regions, and had at least 100 reads in the low-coverage dataset.
[d.] Denotes the number of hg18-pantro2 differences that were not Repeat Masked and did not fall in phastCons regions divided by the total number of positions within the window where differences could have been called (*i.e.* the total number of alignable bases that were not Repeat Masked and did not fall in PhastCons regions).
[e.] Denotes the number of SNPs per window divided by the total number of bases where SNPs could have been called (*i.e.* the total number of alignable bases within the window that were not Repeat Masked and did not fall in phastCons regions that had at least 100 reads).
[f.] Denotes the "# SNPs" divided by d.
