# Supplementary material for: Natural Selection Affects Multiple Aspects of Genetic Variation at Putatively Neutral Sites across the Human Genome
Source: PLoS Genet. 2011 Oct 13;7(10):e1002326. doi: 10.1371/journal.pgen.1002326 (PMC3192825; doi:10.1371/journal.pgen.1002326)
Supplement: Table S2 — Pairwise correlations between variables for the higher-coverage data. (PDF) [file pgen.1002326.s012.pdf]

Table S2: Pairwise correlations between variables for the higher-coverage data.

	Rec. rate	Genic content[a]	GC content[b]	Coverage[c]	d[d]	# SNPs[e]	S_{norm}[f]	π_{norm}[g]	Average MAF	Tajima's D
Rec. rate		-0.0695	0.3808	0.3590	0.2486	0.3094	0.2093	0.2141	0.0865	0.0704
Genic content	4.0E-22		0.1533	0.2653	-0.3101	-0.1683	-0.0330	-0.0442	-0.0435	-0.0451
GC content	0.0	6.9E-102		0.4475	-0.0333	0.1296	0.1451	0.1468	0.0460	0.0372
Coverage	0.0	2.5E-308	0.0		-0.0388	0.0950	0.1260	0.1291	0.0507	0.0350
Divergence	6.1E-270	0.0	3.8E-06	7.0E-08		0.3982	-0.0618	-0.0309	0.0684	0.0677
# SNPs	0.0	1.1E-122	4.0E-73	5.6E-40	0.0		0.8619	0.8146	0.1631	0.1634
S_{norm}	4.1E-190	4.6E-06	2.4E-91	3.4E-69	7.9E-18	0.0		0.9267	0.1451	0.1459
π_{norm}	3.9E-199	8.2E-10	1.7E-93	1.7E-72	1.8E-05	0.0	0.0		0.4617	0.4693
Average MAF	2.3E-33	1.5E-09	1.6E-10	1.8E-12	1.9E-21	3.3E-115	2.1E-91	0.0		0.9809
Tajima's D	1.2E-22	3.6E-10	2.4E-07	1.2E-06	4.9E-21	1.1E-115	2.1E-92	0.0	0.0	

Values of Spearman's ρ for each pair of variables are shown above the diagonal. *P*-values are shown below the diagonal.

[a.] The fraction of each 100 kb window that overlapped with a RefSeq transcript.
[b.] Denotes the number of hg18-pantro2 alignable bases that were not Repeat Masked and did not fall in phastCons regions that were G or C in hg18 divided by the total number of alignable bases within the window that were not Repeat Masked and did not fall in phastCons regions.
[c.] The number of bases per window that were alignable between hg18 and pantro2, were not Repeat Masked, did not fall in phastCons regions, where all six individuals had sequencing data.
[d.] Denotes the number of hg18-pantro2 differences that were not Repeat Masked and did not fall in phastCons regions divided by the total number of positions within the window where differences could have been called (*i.e.* the total number of alignable bases that were not Repeat Masked and did not fall in phastCons regions).
[e.] Denotes the number of SNPs per window divided by the total number of bases where SNPs could have been called (*i.e.* the total number of alignable bases within the window that were not Repeat Masked and did not fall in phastCons regions where all six individuals had sequencing data).
[f.] Denotes the "# SNPs" divided by d.
[g.] Denotes the average number of pairwise differences between sequences within a window divided by d.
