# Supplementary material for: Natural Selection Affects Multiple Aspects of Genetic Variation at Putatively Neutral Sites across the Human Genome
Source: PLoS Genet. 2011 Oct 13;7(10):e1002326. doi: 10.1371/journal.pgen.1002326 (PMC3192825; doi:10.1371/journal.pgen.1002326)
Supplement: Table S4 — Correlation coefficients (Spearman's ) between coding region divergence and neutral diversity (Snorm). (PDF) [file pgen.1002326.s014.pdf]

Table S4: Correlation coefficients (Spearman's ρ) between coding region divergence and neutral diversity (S_{norm})[a]

Dataset	Windows	S_{norm} vs. D_N	S_{norm} vs. D_N; Partial[b]	S_{norm} vs. d_N[c]	S_{norm} vs. d_N; Partial[b,c]	S_{norm} vs. D_S	S_{norm} vs. D_S; Partial[d]	S_{norm} vs. d_S[e]	S_{norm} vs. d_S; Partial[d,e]
Low-coverage	All	0.001	0.023**	0.012	0.036***	-0.005	0.015*	0.023*	0.038***
Low-coverage	Genic	0.014	0.027*	0.007	0.018	0.012	0.019	0.036**	0.034**
Higher-coverage	All	-0.027***	0.004	-0.019	0.018*	-0.026***	0.004	0.017	0.028**
Higher-coverage	Genic	-0.036**	0.015	-0.021	0.011	-0.036**	0.008	0.033**	0.029**
CGS	All	-0.029***	0.015*	-0.017	0.027***	-0.032***	0.008	0.015	0.035***
CGS	Genic	-0.033**	0.022*	-0.021	0.012	-0.037**	0.012	0.031*	0.032**

a. D_N and D_S refer to the number of hg18-pantro2 differences within a window that were not Repeat-Masked and were nonsynonymous and synonymous, respectively. S_{norm} refers to the number of SNPs per window normalized by neutral divergence (d). See the text for a detailed description.

b. Partial correlation controlling for neutral divergence, GC content, recombination rate, and the number of non-Reapeat Masked nonsynonymous sites per window (L_N), and coverage. See the text for a description of these terms.

c. Number of nonsynonymous differences per nonsynonymous site ($d_N = D_N/L_N$).

d. Partial correlation controlling for neutral divergence, GC content, recombination rate, and the number of non-Reapeat Masked synonymous sites per window (L_S), and coverage. See the text for a description of these terms.

e. Number of synonymous differences per synonymous site ($d_S = D_S/L_S$).

* P<0.05
**P<0.01
***P<0.001
