# Supplementary material for: Natural Selection Affects Multiple Aspects of Genetic Variation at Putatively Neutral Sites across the Human Genome
Source: PLoS Genet. 2011 Oct 13;7(10):e1002326. doi: 10.1371/journal.pgen.1002326 (PMC3192825; doi:10.1371/journal.pgen.1002326)
Supplement: Table S5 — Correlation coefficients (Spearman's ) between coding region divergence and neutral diversity (Snorm) for windows in the upper 90th percentile of nonsynonymous divergence per site (dN) or synonymous divergence per site (dS). (PDF) [file pgen.1002326.s015.pdf]

Table S5: Correlation coefficients (Spearman's ρ) between coding region divergence and neutral diversity (S_{norm}) for genic windows in the upper 90th percentile of nonsynonymous divergence per site (d_N) or synonymous divergence per site (d_S)

Dataset	S_{norm} vs. d_N [a]	S_{norm} vs. d_N; partial[b]	S_{norm} vs. d_S [c]	S_{norm} vs. d_S; partial[d]
Low-coverage	-0.064	-0.013	-0.044	-0.084*
Higher-coverage	-0.036	0.000	0.014	-0.062
CGS	-0.083*	-0.081*	-0.008	-0.101*

a. Number of nonsynonymous differences per nonsynonymous site ($d_N = D_N/L_N$).
b. Partial correlation controlling for neutral divergence, GC content, recombination rate, and the number of non-Reapeat Masked nonsynonymous sites per window (L_N), and coverage. See the text for a description of these terms.
c. Number of synonymous differences per synonymous site ($d_S = D_S/L_S$).
d. Partial correlation controlling for neutral divergence, GC content, recombination rate, and the number of non-Reapeat Masked synonymous sites per window (L_S), and coverage. See the text for a description of these terms.

*$0.01<P<0.05$
