# Supplementary material for: Natural Selection Affects Multiple Aspects of Genetic Variation at Putatively Neutral Sites across the Human Genome
Source: PLoS Genet. 2011 Oct 13;7(10):e1002326. doi: 10.1371/journal.pgen.1002326 (PMC3192825; doi:10.1371/journal.pgen.1002326)
Supplement: Table S6 — Selection models used in forward simulations. (PDF) [file pgen.1002326.s016.pdf]

Table S6: Selection models used in forward simulations.

Model	Type of selection	In Figure 2?	Neg. noncoding s[a]	% intronic sites selected	Stength of positive selection (s)[b]	% coding sites positively selected	% windows positively selected (p^+)[c]	% windows with negatively selected introns (p^-)[d]
1	Neutral	Neutral	0	0%	0	0%	0%	0%
2	Neg. coding only	Neg. coding only	0	0%	0	0%	0%	0%
3	Pos. coding only[e]	No	0	0%	3.00×10^{-3}	0.50%	100%	0%
4	Pos. coding only[e]	No	0	0%	6.25×10^{-3}	0.50%	100%	0%
5	Neg & pos. coding	No	0	0%	3.00×10^{-3}	0.50%	100%	0%
6	Neg & pos. coding	First Orange	0	0%	6.25×10^{-3}	0.10%	100%	0%
7	Neg & pos. coding	Second Orange	0	0%	6.25×10^{-3}	0.50%	100%	0%
8	Neg. & pos. coding & neg. noncoding	No	7.5×10^{-5}	25%	6.25×10^{-3}	0.10%	100%	100%
9	Neg & pos. coding & neg. noncoding	First Green	7.5×10^{-5}	25%	6.25×10^{-3}	0.50%	100%	100%

Model	Type of selection	In Figure 2?	Neg. noncoding s[a]	% intronic sites selected	Stength of positive selection (s)[b]	% coding sites positively selected	% windows positively selected (p^+)[c]	% windows with negatively selected introns (p^-)[d]
10	Neg & pos. coding & neg. noncoding [f]	Second Green	7.5×10^{-5}	25%	3.00×10^{-3}	0.50%	10%	90%
11	Neg & pos. coding & neg. noncoding [g]	No	0	0%	3.00×10^{-3}	0.50%	10%	0%
12	Neg & pos. coding & neg. noncoding [h]	No	7.5×10^{-5}	50%	3.00×10^{-3}	0.50%	10%	90%
13	Neg & pos. coding & neg. noncoding [i]	No	0	0%	6.25×10^{-3}	0.50%	5%	0%
14	Neg & pos. coding & neg. noncoding [j]	No	7.5×10^{-5}	50%	6.25×10^{-3}	0.50%	5%[f]	95%
15	Neg. coding & noncoding	First blue	7.5×10^{-5}	25%	0	0%	0%	100%
16	Neg. coding & noncoding	No	1.5×10^{-4}	25%	0	0%	0%	100%
17	Neg. coding & noncoding	No	2.5×10^{-4}	25%	0	0%	0%	100%
18	Neg. coding & noncoding	No	3.75×10^{-4}	25%	0	0%	0%	100%

Model	Type of selection	In Figure 2?	Neg. noncoding s[a]	% intronic sites selected	Stength of positive selection (s)[b]	% coding sites positively selected	% windows positively selected (p^+)[c]	% windows with negatively selected introns (p^-)[d]
19	Neg. coding & noncoding	No	1.00×10^{-3}	25%	0	0%	0%	100%
20	Neg. coding & noncoding	No	5.00×10^{-3}	25%	0	0%	0%	100%
21	Neg. coding & noncoding	No	7.5×10^{-5}	40%	0	0%	0%	100%
22	Neg. coding & noncoding	Second blue	7.5×10^{-5}	50%	0	0%	0%	100%
23	Neg. coding & noncoding	No	7.5×10^{-5}	75%	0	0%	0%	100%
24	Neg. coding & noncoding[k]	No	7.5×10^{-5}	5%	0	0%	0%	100%
25	Neutral with biased gene conversion[l]	No	0	0%	0	0%	0%	0%
26	Neg. coding & noncoding with biased gene conversion[l]	No	7.5×10^{-5}	25%	0	0%	0%	100%

[a.] Strength of negative selection (e.g. selection coefficient) for intronic sites.

[b.] Mean strength of positive selection (e.g. selection coefficient) for the coding sites that were positively selected. Selection coefficients for each positively selected nonsynonymous mutations were drawn from a gamma (shape=(mean*rate), rate=400000) distribution.

[c.] The fraction of the simulated windows where positively selected mutations were allowed to occur on the specified proportion of nonsynonymous mutations. Remaining windows did not include any positive selection.

[d.] The fraction of the windows where negative selection occurred on the specified proportion intronic mutations. Introns evolved neutrally in the remaining windows.

[e.] 0.5% of nonsynonymous mutations were positively selected and the remainder were neutral.

[f.] In 10% of the windows, 0.5% of nonsynonymous mutations were positively selected, and the remainder were negatively selected. In these windows, intronic mutations were neutral. In the remaining 90% of the windows, there was no positive selection, but 25% of intronic mutations were negatively selected.

[g.] In 10% of the windows, 0.5% of nonsynonymous mutations were positively selected, and the remainder were neutral. In these windows, intronic mutations were neutral. In the remaining 90% of the windows, all mutations were neutral.

[h.] In 10% of the windows, 0.5% of nonsynonymous mutations were positively selected, and the remainder were neutral. In these windows, intronic mutations were neutral. In the remaining 90% of the windows, there was no positive selection, but 50% of intronic mutations and all nonsynonymous mutations were negatively selected (where the selection coefficients for the nonsynonymous mutations were drawn from a gamma distribution).

[i.] In 5% of the windows, 0.5% of nonsynonymous mutations were positively selected, and the remainder were neutral. In these windows, intronic mutations were neutral. In the remaining 95% of the windows, all mutations were neutral.

[j.] In 5% of the windows, 0.5% of nonsynonymous mutations were positively selected, and the remainder were neutral. In these windows, intronic mutations were neutral. In the remaining 95% of the windows, there was no positive selection, but 50% of intronic mutations and all nonsynonymous mutations were negatively selected (where the selection coefficients for the nonsynonymous mutations were drawn from a gamma distribution).

[k.] In this model we simulated a 348 kb window (instead of a 100 kb window) with 40 exons (each of size 250 bp) and 38 introns (each of size 7500 bp) with a 53 kb neutral fragment in the center. These simulations show the effect of window size on the correlations between genetic variation and recombination rates.

[l.] This model included biased gene conversion. Here, 1/8 of double-strand breaks resulted in recombination events, while 7/8 were gene conversion events. 51% of the heterozygous AT/GC sites were converted to GC homozygotes. Note, the overall recombination rate for each sequence was increased 8-fold to keep the total number of recombination events the same as in previous simulations. The gene conversion tract length was 500 bp.
