# Supplementary material for: Natural Selection Affects Multiple Aspects of Genetic Variation at Putatively Neutral Sites across the Human Genome
Source: PLoS Genet. 2011 Oct 13;7(10):e1002326. doi: 10.1371/journal.pgen.1002326 (PMC3192825; doi:10.1371/journal.pgen.1002326)
Supplement: Table S7 — Values of Spearman's calculated from forward simulations of various models of selection. (PDF) [file pgen.1002326.s017.pdf]

Table S7: Values of Spearman's ρ calculated from forward simulations of various models of selection.

**Model** [a]	S_{norm} **vs. rec. rate high-coverage** [b]	S_{norm} **vs. rec. rate low-coverage**	**MAF vs. rec. rate high-coverage** [c]	**MAF vs. rec. rate low-coverage**	S_{norm} **vs.** D_{NS} **high-coverage** [d]	S_{norm} **vs.** D_{NS} **low-coverage**	**Divergence. vs. rec. rate low-coverage** [e]
1	0.064 (0.050-0.079)	0.050 (0.036-0.064)	0.030 (0.016-0.044)	0.000 (-0.014-0.015)	-0.019 (-0.032- -0.004)	-0.016 (-0.030 - -0.002)	0.090 (0.076-0.104)
2	0.067 (0.054-0.082)	0.055 (0.040-0.069)	0.048 (0.034-0.062)	0.018 (0.004-0.032)	-0.006 (-0.020-0.006)	-0.008 (-0.022-0.005)	0.124 (0.110-0.138)
3	0.146 (0.133-0.160)	0.125 (0.111-0.139)	0.080 (0.066-0.092)	0.031 (0.017-0.046)	-0.044 (-0.058- -0.030)	-0.040 (-0.054- -0.025)	0.308 (0.296-0.320)
4	0.171 (0.157-0.183)	0.154 (0.140-0.167)	0.107 (0.093-0.120)	0.057 (0.043-0.070)	-0.073 (-0.086 - -0.060)	-0.076 (-0.090 - -0.063)	0.256 (0.244-0.269)
5	0.150 (0.136-0.163)	0.118 (0.105-0.132)	0.091 (0.078-0.105)	0.043 (0.030-0.056)	-0.057 (-0.071- -0.044)	-0.055 (-0.069 - -0.040)	0.308 (0.295-0.320)
6	0.102 (0.088-0.116)	0.083 (0.070-0.098)	0.076 (0.062-0.090)	0.043 (0.029-0.057)	-0.052 (-0.065- -0.038)	-0.048 (-0.063- -0.034)	0.200 (0.187-0.213)
7	0.165 (0.153-0.180)	0.150 (0.137-0.164)	0.103 (0.089-0.117)	0.051 (0.038-0.065)	-0.099 (-0.114- -0.086)	-0.097 (-0.111- -0.084)	0.263 (0.250-0.277)
8	0.165 (0.151-0.178)	0.148 (0.134-0.161)	0.100 (0.082-0.110)	0.049 (0.036-0.064)	-0.027 (-0.042 - -0.013)	-0.031 (-0.046- -0.018)	0.345 (0.332-0.357)
9	0.186 (0.172-0.199)	0.165 (0.153-0.178)	0.112 (0.099-0.124)	0.071 (0.057-0.084)	-0.078 (-0.091- -0.063)	-0.070 (-0.083 - -0.056)	0.296 (0.282-0.308)
10	0.148 (0.135-0.161)	0.121 (0.109-0.134)	0.076 (0.064-0.089)	0.041 (0.030-0.054)	-0.015 (-0.029- -0.002)	-0.016 (-0.030 - -0.003)	0.364 (0.351-0.376)
11	0.076 (0.062-0.089)	0.054 (0.040-0.068)	0.035 (0.022-0.047)	0.002 (-0.010-0.015)	-0.054 (-0.067- -0.041)	-0.048 (-0.061- -0.035)	0.110 (0.098-0.124)
12	0.179 (0.166-0.191)	0.149 (0.136-0.162)	0.089 (0.076-0.102)	0.041 (0.028-0.055)	0.002 (-0.012-0.015)	0.004 (-0.010-0.018)	0.394 (0.383-0.405)
13	0.068 (0.053-0.081)	0.055 (0.40-0.069)	0.032 (0.019-0.045)	0.000 (-0.012-0.014)	-0.060 (-0.073- -0.046)	-0.055 (-0.069- -0.042)	0.096 (0.082-0.110)
14	0.176 (0.163-0.189)	0.156 (0.143-0.168)	0.089 (0.075-0.102)	0.042 (0.028-0.056)	-0.017 (-0.031- -0.004)	-0.018 (-0.032- --0.004)	0.393 (0.382-0.405)

Model [a]	S_{norm} vs. rec. rate high-coverage[b]	S_{norm} vs. rec. rate low-coverage	MAF vs. rec. rate high-coverage[c]	MAF vs. rec. rate low-coverage	S_{norm} vs. D_{NS} high-coverage[d]	S_{norm} vs. D_{NS} low-coverage	Divergence. vs. rec. rate low-coverage[e]
15	0.148<br>(0.134-0.160)	0.125<br>(0.111-0.139)	0.076<br>(0.063-0.090)	0.042<br>(0.028-0.056)	0.006<br>(-0.007-0.020)	-0.002<br>(-0.016-0.012)	0.370<br>(0.358-0.383)
16	0.179<br>(0.166-0.192)	0.162<br>(0.149-0.176)	0.098<br>(0.084-0.112)	0.039<br>(0.025-0.054)	-0.001<br>(-0.016-0.012)	-0.001<br>(-0.014-0.014)	0.414<br>(0.402-0.425)
17	0.228<br>(0.215-0.241)	0.205<br>(0.191-0.219)	0.114<br>(0.100-0.127)	0.061<br>(0.048-0.074)	0.001<br>(-0.013-0.014)	0.000<br>(-0.015-0.013)	0.429<br>(0.418-0.441)
18	0.241<br>(0.228-0.253)	0.214<br>(0.200-0.228)	0.108<br>(0.094-0.120)	0.070<br>(0.055-0.083)	0.012<br>(-0.001-0.027)	0.012<br>(0.00-0.027)	0.359<br>(0.347-0.371)
19	0.149<br>(0.136-0.163)	0.128<br>(0.114-0.142)	0.100<br>(0.087-0.113)	0.051<br>(0.037-0.065)	-0.001<br>(-0.015-0.012)	-0.001<br>(-0.015-0.012)	0.168<br>(0.155-0.182)
20	0.080<br>(0.067-0.094)	0.064<br>(0.050-0.080)	0.050<br>(0.036-0.063)	0.021<br>(0.008-0.035)	0.004<br>(-0.011-0.019)	0.008<br>(-0.006-0.023)	0.120<br>(0.105-0.134)
21	0.172<br>(0.160-0.185)	0.151<br>(0.137-0.164)	0.091<br>(0.077-0.104)	0.063<br>(0.048-0.076)	-0.008<br>(-0.022-0.005)	-0.003<br>(-0.017-0.012)	0.397<br>(0.385-0.408)
22	0.178<br>(0.164-0.192)	0.155<br>(0.142-0.169)	0.089<br>(0.077-0.102)	0.043<br>(0.029-0.055)	0.002<br>(-0.011-0.016)	-0.002<br>(-0.016-0.012)	0.402<br>(0.390-0.413)
23	0.184<br>(0.172-0198)	0.153<br>(0.140-0.167)	0.104<br>(0.091-0.117)	0.059<br>(0.045-0.073)	-0.009<br>(-0.024-0.005)	-0.014<br>(-0.028-0.001)	0.410<br>(0.399-0.421)
24	0.185<br>(0.172-0.198)	0.165<br>(0.151-0.179)	0.090<br>(0.076-0.103)	0.056<br>(0.042-0.070)	-0.004<br>(-0.018-0.010)	-0.007<br>(-0.020-0.007)	0.404<br>(0.392-0.415)
25	0.082<br>(0.068-0.095)	0.064<br>(0.049-0.079)	0.058<br>(0.045-0.072)	0.028<br>(0.013-0.040)	-0.003<br>(-0.017-0.011)	-0.011<br>(-0.024-0.004)	0.111<br>(0.097-0.125)
26	0.165<br>(0.152-0.178)	0.141<br>(0.128-0.155)	0.086<br>(0.073-0.100)	0.040<br>(0.026-0.055)	0.005<br>(-0.010-0.018)	0.011<br>(-0.004-0.025)	0.431<br>(0.420-444)

Values of Spearman's ρ are given in the first line for each model. The second line gives the 95% confidence interval based on bootstrapping the simulation replicates.

[a.] See Table S6 for a description of each model.

[b.] Partial correlation between recombination rate and the number of SNPs/covered base normalized by the number of human-chimp differences in the neutral intergenic part of each simulation replicate controlling for the number of human-chimp differences in the neutral intergenic part of each simulation replicate.
[c.] Correlation between the recombination rate and the average MAF of all the SNPs in the neutral intergenic part of each replicate.
[d.] Partial correlation between the number of human-chimp differences at first and second codon positions per simulation replicate (to represent nonsynonymous divergence) and the number of SNPs/covered base normalized by the number of human-chimp differences in the neutral intergenic part of each simulation replicate controlling for the number of human-chimp differences in the neutral intergenic part of each simulation replicate and recombination rate.
[e.] Correlation between the recombination rate and the number of human-chimp differences in the neutral intergenic part of each replicate.
