# Supplementary material for: Natural Selection Affects Multiple Aspects of Genetic Variation at Putatively Neutral Sites across the Human Genome
Source: PLoS Genet. 2011 Oct 13;7(10):e1002326. doi: 10.1371/journal.pgen.1002326 (PMC3192825; doi:10.1371/journal.pgen.1002326)
Supplement: Table S8 — Pairwise correlations between variables for the CGS data after filtering CpG islands. (PDF) [file pgen.1002326.s018.pdf]

Table S8: Pairwise correlations between variables for the CGS data after filtering CpG islands[a].

| | Rec. rate | Genic content[b] | GC content[c] | Coverage[d] | $d$[e] | # SNPs[f] | $S_{norm}$[g] | $\pi_{norm}$[h] | Average MAF | Tajima's $D$ |
|---|---|---|---|---|---|---|---|---|---|---|
| Rec. rate | | -0.0700 | 0.3884 | 0.2494 | 0.2491 | 0.3011 | 0.2001 | 0.2148 | 0.1012 | 0.0826 |
| Genic content | 1.0E-21 | | 0.1374 | 0.2566 | -0.3116 | -0.1682 | -0.0396 | -0.0472 | -0.0392 | -0.0371 |
| GC content | 0 | 3.1E-79 | | 0.1455 | -0.0170 | 0.0893 | 0.0978 | 0.1085 | 0.0530 | 0.0449 |
| Coverage | 2.5E-262 | 3.1E-278 | 7.9E-89 | | 0.0244 | 0.0871 | 0.0774 | 0.0786 | 0.0214 | 0.0075 |
| Divergence | 9.4E-262 | 0 | 2.0E-2. | 8.4E-4 | | 0.3718 | -0.0640 | -0.0316 | 0.0790 | 0.0732 |
| # SNPs | 0 | 2.2E-118 | 2.4E-34 | 1.0E-32 | 0 | | 0.8746 | 0.8241 | 0.1709 | 0.1567 |
| $S_{norm}$ | 9.9E-168 | 6.3E-08 | 7.3E-41 | 3.5E-26 | 2.1E-18 | 0 | | 0.9236 | 0.1491 | 0.1374 |
| $\pi_{norm}$ | 1.4E-193 | 1.1E-10 | 6.5E-50 | 5.7E-27 | 1.6E-05 | 0 | 0 | | 0.4674 | 0.4656 |
| Average MAF | 1.2E-43 | 8.8E-08 | 4.3E-13 | 3.4E-3 | 3.1E-27 | 3.5E-122 | 3.0E-79 | 0.0 | | 0.9748 |
| Tajima's $D$ | 1.4E-29 | 4.0E-07 | 8.8E-10 | 3.0E-1 | 1.4E-23 | 8.8E-103 | 3.9E-93 | 0.0 | 0.0 | |

Values of Spearman's $\rho$ for each pair of variables are shown above the diagonal. *P*-values are shown below the diagonal.

[a.] CpG islands were defined using the "CpG islands" track on the UCSC Genome Browser.
[b.] The fraction of each 100 kb window that overlapped with a RefSeq transcript.
[c.] Denotes the number of hg18-pantro2 alignable bases that were not Repeat Masked, not in CpG islands, and did not fall in phastCons regions that were G or C in hg18 divided by the total number of alignable bases within the window that were not Repeat Masked, not in CpG islands, and did not fall in phastCons regions.
[d.] The number of bases per window that were alignable between hg18 and pantro2, were not Repeat Masked, not in CpG islands, and did not fall in phastCons regions, where all six individuals had sequencing data.
[e.] Denotes the number of hg18-pantro2 differences that were not Repeat Masked, not in CpG islands, and did not fall in phastCons regions divided by the total number of positions within the window where differences could have been called (*i.e.* the total number of alignable bases that were not Repeat Masked, not in CpG islands, and did not fall in phastCons regions).
[f.] Denotes the number of SNPs per window divided by the total number of bases where SNPs could have been called (*i.e.* the total number of alignable bases within the window that were not Repeat Masked, not in CpG islands, and did not fall in phastCons regions where all six individuals had sequencing data).
[g.] Denotes the "# SNPs" divided by $d$.
[h.] Denotes the average number of pairwise differences between sequences within a window divided by $d$.
